# Resisting decline: the neuroprotective role of resistance exercise in supporting cerebrovascular function and brain health in aging

**DOI:** 10.3389/fphys.2025.1606267

**Published:** 2025-09-22

**Authors:** Elric Y. Allison, Anjali M. Bedi, Aedan J. Rourke, Vanessa Mizzi, Jeremy J. Walsh, Jennifer J. Heisz, Baraa K. Al-Khazraji

**Affiliations:** ^1^ Department of Kinesiology, Faculty of Science, McMaster University, Hamilton, ON, Canada; ^2^ Department of Biomedical engineering, McMaster University , Hamilton, ON, Canada

**Keywords:** resistance exercise, cerebral blood flow (CBF), Alzheimer’s disease related dementias, brain health, cerebrovascular, neurodegeneration, aging, resistance training (RT)

## Abstract

Reduced cerebral blood flow (CBF) and cerebrovascular function are critical early-stage biomarkers preceding changes in brain function and structure observed in normal aging and during the onset and progression of Alzheimer’s Disease and related dementias (ADRD). Though several interventions attempt to curb the effects of aging and brain neurodegeneration, exercise and lifestyle habits remain one of the most impactful and easily modifiable factors for preserving brain health. Although the effects of aerobic exercise on cerebrovascular function and brain health are well established, resistance training (RT) is rapidly increasing in popularity across all age demographics due to its numerous health benefits. Despite the clear physiological benefits of resistance exercise, its potential efficacy for preserving or improving cerebrovascular and overall brain health remains understudied to date. The aim of this review is to examine the literature pertaining to ways in which resistance exercise may reduce the risk of ADRD and slow age-related decline of brain structures and functions. Additionally, this review seeks to highlight key considerations and challenges regarding the feasibility, adoption, and adherence to resistance exercise in the context of normal aging, mild cognitive impairment, and ADRD.

## Graphical Abstract

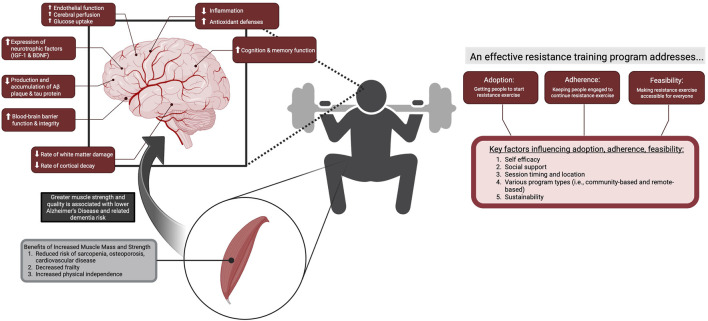

## Introduction

As the global population ages, neurodegenerative conditions like Alzheimer’s disease and related dementias (ADRD) are becoming increasingly prevalent (Rajan et al., 2021). These conditions involve a progressive decline of cognitive function, significant deterioration of neural structures, and widespread metabolic dysfunction throughout the brain (Hoyer et al., 1988; O’Brien J et al., 2020; Zhang B. et al., 2021). Pathological processes such as inflammation, oxidative stress, and cerebrovascular dysfunction emerge in early stages of disease progression (Kinney et al., 2018; Huang et al., 2016; Nelson et al., 2016), long before clinical symptoms like memory loss appear, presenting a significant challenge for the timing and efficacy of targeted pharmaceutical interventions (Iturria-Medina et al., 2016).

Physical inactivity is an important modifiable risk factor for cognitive decline and progression of ADRD. Aerobic exercise is associated with beneficial effects on brain health such that increased physical activity is associated with attenuated rate of age-related cortical atrophy (Colcombe et al., 2003), hippocampal growth (Erickson et al., 2011; ten Brinke et al., 2015), and improved cognitive function (Nagamatsu et al., 2013). Much of the available literature has focused on the protective effects of aerobic exercise and cardiorespiratory fitness in reducing the onset and progression of ADRD (Colcombe et al., 2003; Erickson and Kramer, 2009; Baker et al., 2010; Alty et al., 2020). These neuroprotective and cerebrovascular benefits of aerobic exercise may be partly explained by its ability to reduced systemic inflammation (Gleeson et al., 2011), improved antioxidant defense systems (de Sousa et al., 2017), and improved vascular health and function (Bliss et al., 2021). Converging evidence suggests that reductions in cerebral blood flow (CBF) and cerebrovascular dysfunction may precede structural and functional deficits associated with neurodegeneration, representing one of the earliest indicators of ADRD risk (Jack et al., 2010), and therefore, cerebrovascular dysfunction may serve as an ideal early-stage biomarker that is receptive to aerobic exercise interventions (Kleinloog et al., 2019; Tomoto et al., 2022; Thomas et al., 2020).

Improved skeletal muscle strength is another exercise-induced effect associated with improvements in cognitive function (Van Dam et al., 2018; Frith and Loprinzi, 2018), maintenance of brain structure (Lu et al., 2024; Moon et al., 2019), and reduced risk for onset and progression of ADRD (Boyle et al., 2009; Esteban-Cornejo et al., 2022). Resistance training (RT) has gained popularity, particularly among those prescribing exercise to the elderly, due to its positive effects on muscle strength, metabolism, bone density, and mobility, as well as its potential to reduce the risk and progression of osteoporosis, sarcopenia, frailty, and cardiovascular disease (Westcott, 2012). Likely due to the high magnitude and rapid oscillatory blood pressure responses associated with resistance exercise, the effects of RT on peripheral vascular and cerebrovascular function appear to be distinct from those of aerobic exercise (Thomas et al., 2021). Despite the numerous physiological benefits of RT, its impact on brain health in older adults, particularly those at risk for or living with ADRD, requires further exploration.

Thus, the purpose of this paper is to synthesize the current literature related to the effects of RT on brain health with a specific focus on cerebrovascular function, brain structure, and cognition. This review will first highlight the pathophysiological mechanisms underlying the onset and progression of ADRD. We will also explore mechanisms related to cerebrovascular and neurological adaptations to RT, and their interactions with age- and disease-related changes in cerebrovascular function and brain health. Additionally, this review will identify research gaps and propose future directions throughout for the study of RT and its effects on brain health and ADRD risk and progression. Finally, this review will summarize challenges of prescribing RT interventions (i.e., feasibility, adoption, adherence) in healthy and older adults at risk for ADRD and provide recommendations to overcome these challenges.

## Methods

### Inclusion and exclusion criteria

The population of interest for this review included humans (both male and female) described as middle- or older-aged in the studies, of any health status. We also included mechanistic evidence from young healthy adults and animal studies to help support and interpret findings from human studies when available and appropriate. Studies of any study design were included in this review if they examined the effects of RT. Throughout the manuscript studies involving training interventions refer to the exercise stimulus as RT, whereas acute exercise is referred to as resistance exercise. Acute resistance exercise responses were considered to provide mechanistic background for longer-term adaptations to chronic RT. No limitations related to resistance exercise program design were placed, including factors of intensity, volume, frequency, duration, type of muscular contractions (i.e., concentric, eccentric, isometric), or type of resistance exercise performed. Outcomes of interest for this review included cellular factors such as neurotrophins (i.e., brain derived neurotrophic factor (BDNF), insulin-like growth factor-1 (IGF-1)) and inflammatory markers, hemodynamic and functional measures for any cerebral conduit arteries (e.g., neck artery compliance/stiffness, blood flow, reactivity) and cerebral arteries (e.g., cerebral hemodynamics, blood flow, reactivity, autoregulation), brain structure (regional grey matter integrity, white matter lesions, diffusion tensor images, blood-brain barrier (BBB) integrity), clinical neurodegenerative measures (amyloid deposition, tau neurofibrillary tangles, inflammatory markers), functional connectivity (functional magnetic resonance imaging (fMRI) indices), and cognitive function. Studies were excluded from this review if they did not examine effects of resistance training on any outcomes mentioned above. Additional studies from outside our search including review papers and findings from animal studies are cited for interpretation and speculation of mechanisms underlying effects of resistance exercise on the above-mentioned outcomes.

### Search strategy and screening

Though this review is not a systematic review, we aimed to conduct best research practices and facilitate reproducibility in our search strategy. Our literature search was conducted in 3 databases: MEDLINE, EMBASE, and Web of Science with no date restrictions up to October 2024 (range of publication date for studies: 1985-2024). The search strategy was developed with assistance from a research librarian. For our search we used keywords related to cerebrovascular structure and function, resistance exercise and training, cerebral hemodynamics, cognitive function, brain structure, neurodegeneration, and dementia subtypes. Following the database search, authors (EYA, AMB, AR) screened papers for inclusion eligibility based on the inclusion and exclusion criteria mentioned above. Our search across three databases yielded N = 7275 papers. After screening titles and abstracts for eligibility, there were N = 313 papers for full-text screening. After completion of full-text screening, we had a final pool of N = 172 papers. Because this is not a systematic review, not all 172 of these papers were reviewed comprehensively. Instead, we have used this pool of 172 papers as well as supplementing with articles from outside our search to narratively summarize and review overall trends, relationships, and underlying mechanisms behind the potential efficacy of RT and brain health in older adults and those vulnerable for the onset and progression of vascular dementia and Alzheimer’s disease.

## Metabolic and vascular contributions to ADRD

It is important to recognize that the etiology of ADRD is multifactorial and heterogeneous (Iturria-Medina et al., 2016; Jack et al., 2010; Wang et al., 2017; Habes et al., 2020). In any given case, the onset and progression of ADRD may be driven by one or several interacting mechanisms discussed below, including inflammation and oxidative stress, alterations in cerebral metabolism, cerebral perfusion, systemic and cerebrovascular structure and function, and blood-brain barrier integrity. Moreover, this list is not exhaustive, and ongoing research continues to uncover additional contributors to neurodegeneration, emphasizing the complexity of ADRD pathophysiology and the need for specific prevention and intervention strategies.

### Cerebral metabolism

The brain has limited energy stores and a near exclusive reliance on glucose to support cerebral metabolism that is required to support its extensive energy requirements (Sims-Robinson et al., 2015). Normal aging is accompanied by a decline in cerebral metabolism, driven by impaired mitochondrial ATP production, increased reactive oxygen species (ROS), and decreased antioxidant defenses (Maldonado et al., 2023), and these deficits are amplified in ADRD compared to cognitively normal older adults (Patro et al., 2021; Ionescu-Tucker and Cotman, 2021). Mitochondrial dysfunction, oxidative stress, inflammation, and impaired ATP production occur in a feedforward cycle, serving as both consequences and causes of the marked reduction in glucose metabolism in the brain across the spectrum of ADRD development (Wang et al., 2017). Disrupted glucose metabolism in ADRD is closely linked with an inflammatory and neurotoxic milieu, as increased presence of ROS and pro-inflammatory cytokines reduce expression of glucose transporters, disrupt insulin signalling, and reduce mitochondrial efficiency and function, resulting in impaired transport, uptake, and metabolism of glucose (Rojas‐Gutierrez et al., 2017; Yin et al., 2016). Indeed, glucose hypometabolism is present in both early and advanced stages of ADRD with greater impairments in metabolism coinciding with worsening neurodegeneration (Desgranges et al., 1998; Herholz et al., 2002). Furthermore, impaired cerebral metabolism appears to be an early-stage biomarker for ADRD, as several studies have shown reduced glucose metabolism in the hippocampus and posterior cingulate prior to observation of overt clinical symptoms (i.e., cognitive impairment) (Kumar et al., 2022; Protas et al., 2013; Ferrari et al., 2019; Chen et al., 2021).

### Cerebral perfusion

Converging evidence strongly supports the notion that changes in CBF precede structural and functional deficits associated with neurodegeneration, and may be amongst the earliest indicators of ADRD risk (Jack et al., 2010). In healthy individuals, CBF is tightly coupled to the metabolic demands of neurons (Claassen et al., 2021), however, it is unclear whether reduced CBF in ADRD is a cause or consequence of cerebral hypometabolism. It remains unclear whether the observed cerebral hypoperfusion in those at risk for development of ADRD is primarily a consequence of impairments in systemic vascular health or a result of reductions in in cerebral metabolism. Nonetheless, evidence supports the marked reductions in global CBF in early stages of MCI compared to age-matched controls with normal cognition, and further reduction in CBF as individuals progress from MCI into ADRD (Zhang H. et al., 2021). In people with MCI, the pattern of brain regions with reduced CBF aligns with regions impacted early in the ADRD pathophysiological cascade (posterior cingulate, parietal, temporal, and hippocampal regions (Zhang H. et al., 2021), supporting a role for cerebral hypoperfusion in the MCI-ADRD progression.

Cardiovascular disease is also associated with development of neurodegenerative conditions, driven by both cardiac insufficiency-related hypoperfusion as well as damage to cerebral microvasculature, similarly reducing blood flow to cerebral tissues (Newman et al., 2005; Stefanidis et al., 2018; KNOPMAN, 2007). The consequences of the cardiovascular disease-related reductions in CBF include accelerated reduction in frontal, parietal, and temporal grey matter volumes (Anazodo et al., 2013), greater white matter hyperintensity burden (Moroni et al., 2018), cognitive deficits (Jiang et al., 2023), and increased risk of ADRD (Brain et al., 2023). Given the role of CBF in supporting neuronal function, cardiovascular disease appears to exacerbate the feed-forward processes in neurodegeneration by contributing to chronic hypoperfusion and metabolic stress in these vulnerable regions. In the context of ADRD risk, cerebral hypoperfusion also appears to precede Amyloid-β (Aβ) and hyperphosphorylated tau accumulation in the ADRD pathophysiological cascade (Iturria-Medina et al., 2016), particularly in regions vulnerable to early pathology (entorhinal cortex, hippocampus, inferior parietal cortex, inferior temporal cortex) (Mattsson et al., 2014). Increased Aβ and neurofibrillary tangle deposition in the brain subsequently promote further cerebrovascular dysfunction via increased ROS production and inflammation (Alavi Naini and Soussi-Yanicostas, 2015; Cheignon et al., 2018). Thus, the literature related to the chronology of ADRD pathophysiology implicates CBF as a critical early-stage target for attenuating neurodegenerative cascades prior to symptomatic changes in brain function.

### Systemic vascular structure and function

Though it remains unclear whether reductions in CBF or cerebral metabolism occurs first in the ADRD cascade, mechanical factors such as systemic and cerebral arterial stiffening are additional early stage vascular markers implicated in cerebral hypoperfusion in older adulthood beyond the typical effects of aging (Mitchell, 2011; Jefferson et al., 2018; Muhire et al., 2019). The processes leading to increased arterial stiffness are related to structural remodelling such as increased collagen and decreased elastin in the vessel wall, as well as higher resting vascular tone, endothelial dysfunction, and reduced nitric oxide bioavailability (Lyle and Raaz, 2017). Other cellular factors such as oxidative stress and inflammation can also negatively impact systemic and cerebral endothelial function by damaging endothelial cells (Shaito et al., 2022) and reducing nitric oxide bioavailability (Gryglewski et al., 1986). Long-term consequences of arterial stiffening include negative impacts on brain function, BBB breakdown (Krizbai et al., 2005), reduced CBF, and blunted cerebrovascular reactivity (Lourenço et al., 2017). Increased arterial stiffness in cerebral conduit arteries impairs their ability to dampen blood flow ejected from the heart, leading to transmission of high pressure and pulsatile blood flow to the cerebral microvasculature and neuronal tissue. This increased transmission of pulsatile and high-pressure blood flow into the cerebral circulation is associated with cerebral microvascular damage, which contributes to chronic reductions in CBF seen with aging and disease (Suri et al., 2020). Cerebrovascular pulsatility is elevated in ADRD compared to healthy controls (Chung et al., 2017; Roher et al., 2011). Previous work from our group supports the importance of arterial stiffness as an early-stage biomarker for future decrements in brain structure by showing that high levels of arterial stiffness in midlife, but not age-related arterial stiffening, are predictive of lower grey matter volume and greater white matter hyperintensity burden in a 10-year follow-up (Allison and Al-Khazraji, 2024a). Bown et al similarly showed strong predictive effects of baseline arterial stiffness on occipital and hippocampal volume and temporal lobe white matter hyperintensity volume in a 5-year follow up (Bown et al., 2021), all of which are brain regions that are highly vulnerable to neurodegeneration (Fjell et al., 2014). These findings emphasize the importance of systemic vascular function and mechanics on long term brain health, likely mediated by damage and mechanical stress on cerebral microvasculature.

### Cerebrovascular function

Optimal function of cerebral conduit arteries is another critical factor in ensuring CBF matches the metabolic demands for brain function and maintain long-term brain health. Several techniques can be used to assess cerebral arterial function, including measures of perfusion (middle cerebral artery blood velocity; MCAv, CBF), cerebrovascular reactivity, cerebrovascular conductance, cerebrovascular resistance, cerebrovascular pulsatility, cerebral autoregulation, and neurovascular coupling (Ozturk and Tan, 2018). MCAv begins to decrease in early midlife (Alwatban et al., 2021), aligning with the reductions in global CBF observed with aging (Chen et al., 2011). Compared to healthy older adults, MCAv is lower in age-matched individuals with MCI and ADRD, worsening along the neurodegenerative spectrum (Zhang H. et al., 2021; Sabayan et al., 2012). Cerebrovascular resistance can also provide information on cerebrovascular function in ADRD. Greater cerebrovascular resistance is indicative of constricted downstream vessels (higher vascular tone) in the cerebral circulation, restricting perfusion into cerebral tissues (Claassen et al., 2021). Compared to cognitively normal older adults, cerebrovascular resistance is higher in individuals with MCI and ADRD, and has been associated with greater Aβ burden, cortical atrophy, and risk of progression to ADRD (Yew and Nation, 2017). Additionally, increased cerebrovascular resistance has been suggested to precede observable reductions in regional CBF (Yew and Nation, 2017). Higher resting cerebrovascular tone may occur due to endothelial dysfunction (Bolduc et al., 2013) or chronic overactivity of the sympathetic nervous system (Willie et al., 2014), as seen in cardiovascular disease (Jay Widmer and Lerman, 2014; Malpas, 2010) and diabetes (Calles-Escandon and Cipolla, 2001; Esler et al., 2001), conditions which contribute to ADRD risk.

Cerebrovascular resistance serves as the foundational basis other indices of cerebrovascular function and health. Cerebrovascular reactivity is a frequently used measure to provide insight into the health and function of the cerebral vasculature, and is dictated by changes in cerebral vascular tone in response to a given stimuli (Claassen et al., 2021). Assessing changes in either large cerebral artery cross-sectional area and CBF (MRI) or cerebral hemodynamics (MCAv) in response to a vasoactive stimuli, such as carbon dioxide (CO_2_), forms the basis of cerebrovascular reactivity (Wang et al., 2023). CO_2_ is a potent regulator of CBF, as elevated CO_2_ and the accompanying pH change causes healthy cerebral arterioles to dilate, subsequently increasing conductance and CBF (Hoiland et al., 2019). Crucially, individuals in ADRD demonstrate marked impairments in cerebrovascular reactivity to CO_2_ compared to controls (Glodzik et al., 2013; Alwatban et al., 2019; Gao et al., 2013; Vicenzini et al., 2007). Based on these associations, cerebrovascular reactivity may relate to early cerebrovascular dysfunction associated with neurodegenerative diseases like ADRD. Another key regulator of CBF is neuronal activity, mediated through the process of neurovascular coupling (Phillips et al., 2016). Briefly, neurovascular coupling describes the change in cerebral oxygen extraction or blood flow delivery to meet the metabolic demands of active neurons (Phillips et al., 2016). Similar to cerebrovascular reactivity, impaired neurovascular coupling is attenuated with advanced age (Peng et al., 2018), MCI and ADRD (Phillips et al., 2016; Peng et al., 2018; Nicolakakis and Hamel, 2011). Factors associated with impaired cerebrovascular reactivity and neurovascular coupling include cerebral microvascular dysfunction, insulin-like growth factor 1 (IGF-1) deficiency, and oxidative stress-related inhibition of neuronal and astrocytic vasodilators (Phillips et al., 2016; Zlokovic, 2005). The importance of these cerebrovascular functional measures are supported by the graded relationship between impairment in cerebrovascular reactivity to CO_2_ (Kim et al., 2021; Silvestrini et al., 2006) and neurovascular coupling responses and severity of cognitive impairment in individuals with MCI and ADRD (van Dijk et al., 2024). Overall, these findings emphasize the importance of cerebral vasoreactivity as a marker of cerebrovascular and overall brain health.

### Cerebral autoregulation

The responsiveness of cerebral vasculature to stimuli also underlies cerebral autoregulation. The concept of cerebral autoregulation describes the ability of the cerebral vasculature to regulate CBF during oscillations in cerebral perfusion pressure (Claassen et al., 2021). Currently the data suggests that autoregulatory function remains intact in individuals across the spectrum of ADRD progression (Claassen et al., 2021; Claassen and Zhang, 2011; de Heus et al., 2018; de Jong et al., 2019). Increased resting cerebrovascular tone in individuals along the ADRD progression spectrum (Yew and Nation, 2017) is likely to restrict transient increases in blood pressure into the cerebral circulation. Thus, an absence of a change in CBF during increases in blood pressure due to increased resting cerebrovascular resistance in neurodegenerative models may mask differences in autoregulatory function. Whether blood pressure oscillation direction (increases vs. decreases) affects interpretation of data pertaining to autoregulatory function in cognitive decline, vascular dementia, and ADRD remains unclear.

### Blood-brain barrier (BBB)

The structural and functional integrity of the BBB is emerging as another early-stage biomarker for neurodegenerative conditions. A critical neuroprotective structure, the BBB’s primary role is regulating the transport of compounds into (influx) and out of (efflux) the parenchyma (Moyaert et al., 2023). A key characteristic of the BBB compared to peripheral vasculature is the arrangement of endothelial cells, which include tightly sealed cell-to-cell contacts called tight junctions (Moyaert et al., 2023). The BBB, similar to the peripheral vasculature, is susceptible to degradation and dysfunction of endothelial cells through oxidative stress, inflammation, and mechanical hemodynamic stress (i.e., high systolic pressures and pulsatility) (Moyaert et al., 2023; de Montgolfier et al., 2019). Damage to the tight endothelial junctions results in increased BBB permeability, leading to the entry of immune cells and inflammatory cytokines into the parenchyma, as well as impaired clearance of metabolic waste and neurotoxic compounds (Zlokovic, 2004; De Boer and Gaillard, 2006). One of the most vulnerable brain regions to normal age-related and disease related alterations in BBB permeability is the hippocampus (Sweeney et al., 2018a; Sweeney et al., 2018b). The disruption in BBB in the hippocampus is accelerated with MCI and ADRD progression compared to normal aging (Sweeney et al., 2018b). BBB dysregulation precedes Aβ and tau protein deposition, cognitive decline, and structural changes in brain tissue (Iturria-Medina et al., 2016). The role of the BBB in the progression of ADRD is further supported by evidence showing that disrupted BBB function impairs efflux of Aβ from the parenchyma (Wang et al., 2017; Zlokovic, 2004; Sweeney et al., 2018b). However, with continued accumulation of Aβ plaques, the inflammatory and neurotoxic parenchymal environment can progress to further damage the BBB (Erickson and Banks, 2013). This cycle of Aβ accumulation causing BBB damage, leading to further impairments in Aβ efflux and thus greater accumulation is a major process underlying the severe and rapid loss of function in AD. Thus, the BBB may represent a viable therapeutic target to prevent or attenuate progression of ADRD prior to histopathological, structural, or functional changes in the brain.

## Why resistance exercise?

### Muscle strength as a risk factor for ADRD

Muscle weakness and the associated frailty are associated with MCI and ADRD severity (Moon et al., 2019; Boyle et al., 2009; Filardi et al., 2022; Dost et al., 2023). Sarcopenia and the accompanying loss in muscle strength and balance leads to frailty, decreased independence, lower rates of physical activity, and increased sedentary time (Loprinzi, 2016). Importantly, patterns of age-related muscle loss appear to be different between sexes, with men demonstrating a greater rate of relative loss in muscle mass with age and females having a greater prevalence for sarcopenia–likely due to males having a higher muscular reserve compared to females (de Jong et al., 2023). The relationship between sex and prevalence of sarcopenia may partially be explained by longer life expectancy in females–as it is primarily an aging related disorder. Nonetheless, the greater likelihood of sarcopenia in females aligns with epidemiological studies that point to females comprising two thirds of all ADRD diagnoses (Mielke, 2018; Alzheimer’s Association, 2019). Improving skeletal muscle mass and quality via RT may therefore be a critical non-pharmacological approach to attenuate systemic inflammation and ROS production associated with sarcopenia sedentarism (Meng and Yu, 2010; Dalle et al., 2017; Pan et al., 2021; Laufs et al., 2005; Gratas-Delamarche et al., 2014), and slow the rate of age and disease-related neurodegeneration. Given its beneficial effects on muscle size and quality (Westcott, 2012), and improved metabolic function (i.e., improved glucose metabolism, insulin sensitivity) (Zhou et al., 2022), partaking in regular RT is a promising behaviour to attenuate cellular and behavioural risk factors for neurodegenerative processes.

### Resistance exercise and sleep

Sleep duration and quality are important factors in the development and progression of ADRD (Himali et al., 2023). The glymphatic system, most active during slow-wave or restorative sleep, is essential for facilitating clearance of metabolic waste (Reddy and van der Werf, 2020; Xie et al., 2013). This process of glymphatic clearance during sleep includes the efflux of Aβ and tau proteins out of the parenchyma (Xie et al., 2013; Holth et al., 2019; Kang et al., 2009). Beyond the two primary proteins of focus in ADRD progression, glymphatic clearance also helps support removal of inflammatory markers to maintain the parenchymal environment and reduce neurotoxic load on neurons (Mogensen et al., 2021). Meta-analyses have shown that habitual participation in RT is associated with improved sleep quality (Kovacevic et al., 2018). Further, in older sarcopenic adults, a 12-week RT program (3 times per week of full body RT at 75% 1RM) was shown to reduce sleep latency (shorter time necessary to fall asleep) as well as increase time spent in slow-wave sleep, when a large proportion of glymphatic drainage and restorative processes occur (De Sá Souza et al., 2022). While more work is needed to determine the optimal parameters of RT programs for improving sleep, it is nonetheless important to recognize the role of RT, and exercise in general, as a non-pharmacological strategy to support brain health. RT may help improve sleep, which could mitigate the accumulation of metabolic waste, inflammatory markers, and neurotoxic proteins, ultimately lowering the risk for the onset and progression of ADRD.

### Resistance exercise as a unique hemodynamic stressor

From a vascular perspective, the hemodynamic stimulus associated with resistance exercise, particularly at high intensities is unique, characterized by high magnitude and rapid oscillations in blood pressure (Perry and Lucas, 2021). These oscillations in blood pressure involve a transition between hyper- and hypotension (corresponding to concentric and eccentric muscular contractions) within each repetition cycle (Perry and Lucas, 2021). While more research is necessary, repeated exposure to oscillatory hemodynamic stress and the associated patterns of shear stress on the endothelium may prime the vasculature to better handle both surges and sudden drops in systemic blood pressure, potentially leading to better maintenance and regulation of CBF during pressure challenges. Improved regulation of CBF in response to both transient hyper- and hypotension subsequently protects cerebral microvasculature from hyperperfusive injury and damage as well as ensuring cerebral perfusion is able to match metabolic demand during periods of reduced driving pressures (i.e., orthostatic stress) (Claassen et al., 2021). Indeed, recent cross-sectional evidence supports enhanced pressure regulation in the brain in resistance-trained individuals (Korad et al., 2024; Roy et al., 2022). Thus, the rationale for RT interventions to mitigate neurodegenerative processes is strengthened by the distinct vascular and cerebrovascular adaptations (Allison and Al-Khazraji, 2024b; Zhang Y. et al., 2021), as well as the apparent neuroanatomical and cognitive benefits that will be discussed in subsequent sections of this review.

### Resistance exercise as a cognitive stimulus

Resistance exercise involves multiple aspects that require attention and focus. These aspects include neuronal adaptations centered around synchronized recruitment of motor neurons in several different muscle groups, matching muscle fibre recruitment to exercise intensity, learning new motor patterns involving multiple muscle groups or muscles in isolation (Carroll et al., 2001; Gabriel et al., 2006), keeping track of repetitions and sets completed, and use of intentional breathing techniques such as the Valsalva Maneuver (Forte et al., 2013; Lepley and Hatzel, 2010). Indeed, motor task complexity has been linked to neuroplasticity (Carey et al., 2005). Thus, while significantly more research is necessary, the novelty and complexity of resistance exercises may be an important factor underlying its pro-cognitive effects and efficacy in driving neuroplasticity. Nonetheless, resistance exercise is a unique model, likely eliciting different neuronal and physiological adaptations related to improved brain health and function compared to other forms of exercise. The mechanisms underlying protective effects of RT on the brain and its role in reducing risk of neurodegeneration are discussed herein.

An important concept to define prior to synthesis of literature is progressive RT. Briefly, progressive RT relies on a fundamental principle of resistance exercise prescription–progressive overload in which intensities, reps, or sets may start lower but increase gradually over the duration of the intervention (Kraemer et al., 2002). The fundamental principles of progressive overload are rooted in facilitating continued strength gains; however, given the relationship between muscular strength and overall brain health, there may be potential application of these principles in improving brain health. Progressive overload is applied in both human and animal research and will be referred to as progressive RT herein.

## Effects of resistance training on brain-related outcomes

The issue of maintaining and promoting brain health is highly complex. However, multiple factors, including inflammation (Ahmad et al., 2022) and cerebral perfusion (Wolters et al., 2017) have been supported as early-stage biomarkers for neurodegenerative processes that are receptive to intervention prior to onset of clinically relevant loss in brain function. Additionally, neurotrophic factors such as IGF-1 and BDNF are critical targets for facilitating slower rates of structural and functional decline in the brain with advancing age–as both promote neurogenesis (Park and Poo, 2013; Anderson et al., 2002), as well as neuronal plasticity (Zagrebelsky and Korte, 2014) and survival (Park and Poo, 2013; Nieto-Bona et al., 1997). While the factors underlying brain health and longevity discussed below are not exhaustive, they represent what appear to be the most quantifiable and targetable factors underlying neurodegenerative processes along the MCI-ADRD continuum. Below we discuss the effects of resistance exercise on both humans and in animal models. Though findings from animal models provide an important basis for future research in humans, the findings from studies using animal models need to be interpreted with caution and as hypothesis generating, rather than definitive evidence of the neuroprotective effects of RT. Nonetheless, for completeness and interpretive purposes, studies using animal models are included in this review and are explicitly described as such herein.

### Effects of resistance exercise training on oxidative stress and inflammation

Chronically elevated oxidative stress and diminished antioxidant defenses causes neuronal damage and mitochondrial dysfunction, which play key roles in neurodegenerative pathologies (Guo et al., 2013). The brain is particularly susceptible to oxidative stress given its limited antioxidant enzymatic capacity to counteract reactive oxygen species production (Ferreira et al., 2015). Despite a well-established relationship between acute resistance exercise and increased oxidative stress (Fisher-Wellman and Bloomer, 2009), habitual RT has been associated with a reduction in markers of oxidative stress at rest, with the bulk of evidence coming from animal models. 6 weeks of progressive ladder-climbing RT (8 climbs, progression from 50% to 100% maximal carrying load (MCL), 3 times per week) mitigates the rise in reactive oxygen species production, as represented by lipid peroxidation/malondialdehyde levels in the brain tissue of an AD rodent model (Özbeyli et al., 2017). The RT intervention also improved antioxidant defence capabilities, as represented by glutathione levels (Özbeyli et al., 2017). Interestingly, the same results were not observed in healthy age-matched mice, as 6 weeks of progressive ladder-climbing RT (3 climbs, progression from 50% to 100%MCL, 5 times per week) decreased lipid peroxidation in the cerebral cortex but increased lipid peroxidation in the hippocampus (Feter et al., 2019). It is possible that RT in healthy mice may initially act on antioxidant defence capacity, rather than mitigating reactive oxygen species production directly. The benefits of RT on oxidative stress in rodent models of AD do appear to persist beyond 6 weeks, as another trial found that 8 weeks of ladder-climbing RT (8 climbs, 2 repetitions at 50%, 75%, 90%, and 100% MCL, 3 times per week) prevented a rise in lipid peroxidation (Schimidt et al., 2019). This evidence suggests that RT may be more effective at reducing oxidative stress in situations where ROS and antioxidant balance is compromised, as observed along the continuum of neurodegenerative disease progression or with aging. Indeed, 24 weeks of moderate intensity RT (3 sets of 8–12 reps with progression to 12-16 reps at 65%–75% 1RM, 2 times per week) in combination with cognitive stimulation and aerobic exercise in older adults with MCI reduced plasma markers of lipid peroxidation (Rondão et al., 2022). Similarly, 6 months of resistance training improved numerous blood indices of oxidative stress including reduced oxidative damage, ameliorated antioxidant defence and DNA stability in older adults (Franzke et al., 2018). Thus, current evidence suggests that RT protocols are beneficial for mitigating rises in oxidative stress that occur along the development of neurodegenerative diseases and aging. Future work is required to determine if this training modality can bolster antioxidant defenses and mitigate excess oxidative stress in healthy human populations.

Inflammation and oxidative stress are closely interconnected, as the cellular damage imposed by increases in oxidative stress prompts immune inflammatory responses which contribute to the development and severity of neurodegeneration (Heneka et al., 2015). Despite acute physical exercise transiently increasing inflammatory responses, longer-term RT lowers circulating levels and cellular concentrations of anti-inflammatory cytokines. Acute long duration resistance exercise (180 min of bilateral knee extensions at 55% 1RM) in elderly populations is typically accompanied by an acute inflammatory immune response characterized by the release of interleukin-6 (IL-6) into the circulation (Steensberg et al., 2002). Whereas, 4 weeks of progressive ladder-climbing RT (2 climbs per session at 75%, 90%, and 100% MCL) 5 times per week in a mouse model of AD lowered circulating IL-6 to levels comparable to healthy controls (Hashiguchi et al., 2020). Similarly, 12 weeks of progressive full body RT 3 times per week (3 sets of 10 reps, progression from 50%–80% 1RM), in older humans lowers basal IL-6 levels, which may represent an adaptation to the exercise (Forti et al., 2014). Though IL-6 has pro-inflammatory functions, it can also stimulate the release of IL-10 (Ostrowski et al., 1999) which in turn inhibits the production of inflammatory cytokines such as IL-1β, and tumour necrosis factor-α (TNF-α) (Cassatella et al., 1993). Indeed, progressive RT (15 climbs, progression from 15%–75% of MCL over 4 weeks) performed on alternating days attenuated neuroinflammation in the frontal cortex and hippocampus of mice by reducing mRNA expression of TNF-α and protein concentrations of IL-1β (Liu et al., 2020). In contrast to these findings, 8 weeks of progressive RT (3 sets of 8–12 climbs, 50%–100% of MCL) on alternating days in aging rats observed increased hippocampal protein expression of TNF-α and IL-1β (Vilela et al., 2017). However, given that this study did not measure IL-6 or IL-10, it is unclear where there was also a concomitant increase in anti-inflammatory molecular factors to counteract this response. Overall, current research presents substantial evidence for the therapeutic potential of RT to mitigate oxidative stress and inflammation, which are crucial pathophysiological factors underlying neurodegenerative disease pathogenesis (Vints et al., 2024).

### Effects of resistance exercise training on ADRD biomarkers

For the last 2 decades, the discourse related to the underlying mechanism for development of AD specifically has largely focused on the amyloid hypothesis, attributing the transition from MCI to AD to the accelerated rate of Aβ plaque deposition and subsequent neurofibrillary tangle formation (Hardy and Higgins, 1992). ROS production and inflammation are the assumed to be the primary drivers of accelerated Aβ plaque deposition and neurofibrillary tangle formation (Huang et al., 2016; Ionescu-Tucker and Cotman, 2021; Cheignon et al., 2018). Several animal studies have shown proof-of-concept for alleviation of amyloid burden with RT interventions. In AD animal models, 3 days of progressive ladder-climbing RT (6–11 climbs progressing from 75%–100% MCL) on alternating days for 4 weeks resulted in significant reductions in Aβ levels and significant reductions in IL-1, IL-4, IL-6, and no differences in IL-10 between exercising and AD non-exercising controls (Hashiguchi et al., 2020). The mechanisms for reduced Aβ are hypothesized to be related to restoration of the BBB, facilitating efflux of Aβ from the parenchyma (Zlokovic, 2004). A separate study from the same research group, using the same exercise protocol similarly showed significant reductions in Aβ levels and increased microglia in the hippocampus in AD mice (Campos et al., 2023). These findings are supported by work from Liu et al who similarly observed increased expression of synaptic signalling proteins in the hippocampus, and reductions in Aβ and tau burden in the frontal cortex and hippocampus of AD mice with 4-week progressive ladder-climbing RT (15 climbs, progression from 15%–75% MCL) (Liu et al., 2020).

Özbeyli et al showed that 3 days of progressive ladder-climbing RT (50%–100% MCL) per week for 6 weeks significantly reduced amyloid burden in AD mice compared to sedentary and aerobic trained AD mice (Özbeyli et al., 2017). In animal models of AD, RT has shown efficacy for mitigating accumulation of Aβ plaques, with fewer studies showing positive effects on neurofibrillary tangles. Exercise related attenuations in Aβ production is a critical consideration as well. Work from Marko et al demonstrated that treatment of human neuronal cells with post-exercise serum, albeit aerobic, was associated with decreased expression of amyloid processing enzyme β-secretase linked to production of Aβ and increased expression of non-amyloidogenic enzyme α-secretase, producing the soluble amyloid proteins that have not been implicated in neurodegeneration (Marko et al., 2022). The implications of the work from Marko et al should not be understated, as it provides critical insight into the effectiveness of even a single bout of exercise on reducing Aβ production and plaque accumulation. Future studies should aim to extend these results to determine whether RT would have similar anti-amyloidogenic effects on the amyloid precursor protein processing. In addition to acute effects, future studies should also aim to investigate the effects of RT interventions on biomarkers of ADRD in humans to continue to build understanding on how exercise can attenuate or potentially reverse ADRD pathology.

### Effects of resistance exercise training on circulating neurotrophins

Acute and chronic changes in circulating neurotrophic growth factors following RT have been extensively studied as potential mechanisms that contribute to the structural and functional improvements in brain health with RT. Although RT increases several circulating growth factors that are important for brain health, this review will primarily focus on BDNF and IGF-1, given their prominent role in modulating brain plasticity. BDNF is a member of the neurotrophin family that contributes to a myriad of functions including synaptic plasticity (Zagrebelsky and Korte, 2014), neuronal growth, survival, and repair (Park and Poo, 2013), and is essential for facilitating learning and memory (Cirulli et al., 2004; Lu et al., 2014). In particular, BDNF has been identified as a prominent contributor to exercise-induced changes in brain health. Evidence from murine models demonstrate that hippocampal and cortical BDNF mRNA expression remains elevated for up to 24 h post-exercise, supporting the role of BDNF in facilitating changes in brain function well beyond the cessation of exercise (Rasmussen et al., 2009). Further research has also provided evidence of increased hippocampal BDNF following both acute resistance exercise in insulin-resistant rats (Berbert-Gomes et al., 2024) and increases in basal BDNF concentrations following 8 weeks of progressive ladder-climbing RT (8 climbs, 2 repetitions for each load of 50%, 75%, 90%, and 100% MCL, 3 times per week) in a rodent model of AD (Jafarzadeh et al., 2021). Collectively, this evidence suggests that BDNF concentrations are rapidly increased following acute resistance exercise and performing repeated bouts of resistance exercise (i.e., RT) may lead to sustained increases in resting BDNF over time.

Given that blood BDNF concentrations decline with aging (Lommatzsch et al., 2005; Ziegenhorn et al., 2007), extensive research has aimed to assess whether RT is an effective lifestyle intervention to help mitigate these reductions in humans. Research assessing exercise-induced BDNF responses in humans are limited to only measuring peripheral concentrations of blood BDNF, unlike in animal models where brain BDNF expression and/or concentrations can be directly measured. As a result, it is difficult to precisely localize potential changes in brain function arising from exercise-induced increases in BDNF. Nonetheless, there is evidence to suggest that peripheral BDNF may cross the BBB into the brain parenchyma (Pan et al., 1998) and circulating concentrations closely mirror BDNF content in the brain (Klein et al., 2011; Sartorius et al., 2009), suggesting that peripheral BDNF concentrations may be a viable surrogate for brain BDNF concentrations. Numerous research groups have shown that acute resistance exercise robustly increases circulating BDNF in humans, albeit transiently (Yarrow et al., 2010; Walsh et al., 2015; Marston et al., 2017; Church et al., 2016), while other studies have demonstrated no such change in circulating BDNF following acute resistance exercise (Correia et al., 2010; Goekint et al., 2010). Though the circulating BDNF response to acute resistance exercise is transient, Walsh et al demonstrated that post-exercise BDNF release persists, but did not increase as a result of 8 weeks of RT (3 exercises, 4 sets, 8-12 repetitions, progression from 60%–80% 1RM, 3 times per week (Walsh et al., 2015). In contrast, Church et al, who found that independent of training structure (high volume-low intensity [10–12 reps; 70% 1RM] vs. high intensity-low volume [3-5 reps; 90% 1RM], 4 days per week for 8 weeks) the magnitude of post-exercise BDNF release was increased compared to pre-training levels (Church et al., 2016), suggesting that each bout of resistance exercise session independent of training status provides a “dose” of BDNF to the brain, and the release of BDNF can be increased with habitual RT. These acute increases in circulating BDNF from resistance exercise may be an important stimulus for longer-term adaptations to brain health, given the plethora of evidence supporting the beneficial effects of RT on cognitive function, especially executive function (Liu-Ambrose et al., 2010; Landrigan et al., 2020). The neurocognitive effects of repeated acute BDNF doses via RT requires further research, as much of the current evidence aside from the work conducted by Church et al suggests that RT does not increase basal blood BDNF levels. Progressive RT interventions in middle-aged to older adults for 8 weeks (Walsh et al., 2015; Ruiz et al., 2015), 12 weeks (Forti et al., 2014), and as long as 9 months in individuals with type 2 diabetes (Swift et al., 2012) have not been shown to change basal serum BDNF. Previous work has also demonstrated that 8 weeks of combined aerobic and RT (full body RT; 3 sets of 8–15 reps to failure, 3 times per week) does not change basal plasma BDNF levels in individuals with Type 2 Diabetes (Silveira-Rodrigues et al., 2023) and 24 weeks of multicomponent exercise decreases basal plasma BDNF in older adults with MCI (Rondão et al., 2022). Contrarily, other work has shown that 10 weeks of RT (1-3 sets of 8 repetitions at 75% 1RM, 3 times per week) (Coelho et al., 2012; Pereira et al., 2013) and 16 weeks of a multicomponent exercise paradigm (2 sets of 6-8 repetitions, 2 times per week) (Vaughan et al., 2014) were both sufficient to increase basal plasma BDNF in otherwise healthy elderly adults. In summary, a single bout of resistance exercise likely leads to robust and transient increases in circulating BDNF, which may be leveraged in chronic RT interventions with repeated bouts to improve and/or protect brain health with aging. Furthermore, the literature related to the effects of RT on basal BDNF remain mixed and requires further investigation to clarify this relationship, with an emphasis on delineating these findings based on populations (i.e., young vs. older adults, healthy vs. chronic health conditions such as diabetes, ADRD) and exercise parameters (i.e., multicomponent vs. RT alone, intensity, frequency, duration of training).

Liver-derived IGF-1 is another neurotrophic growth factor implicated in long-term brain health. Similar to BDNF, IGF-1 modulates exercise-induced neurogenesis (Anderson et al., 2002), synaptic density and plasticity (Nieto-Bona et al., 1997), and hippocampal expression of BDNF (Ding et al., 2006). Furthermore, IGF-1 may regulate Aβ plaque concentrations, as lower circulating IGF-1 have been associated with Aβ plaque accumulation in the rodent brain (Carro et al., 2002). IGF-1 deficiencies are also implicated in the development of neurodegenerative diseases such as AD (Westwood et al., 2014), while excess levels of IGF-1 are associated with aberrant cellular growth (i.e., cancers) (Qian and Huo, 2020). The focus of this section is to review evidence related the impacts of RT-induced IGF-1 on brain health and not to imply that more circulating IGF-1 is better. Indeed, in normal physiological circumstances, basal circulating IGF-1 is tightly regulated by the cerebral spinal fluid (CSF) and any changes in circulating IGF-1 due to a single bout of resistance exercise are transient. Limited evidence of basal changes in IGF-1 following a period of RT training are likely due to corrections of a deficit, rather than an increase in IGF-1 per se. Evidence from animal models suggest that improvements in spatial memory following an acute bout of resistance exercise may be mediated via IGF-1, however the relationship between IGF-1 and short term cognitive performance in humans remains unclear (Cassilhas et al., 2012). Findings from human trials showed that a single bout of isometric knee extension resistance exercise (40% and 110% of maximal effort) (Vega et al., 2010) elicits an increase in circulating serum IGF-1 in young adults, similar to both moderate (50% 1RM) and high-intensity (80% 1RM) resistance exercise (Tsai et al., 2014). The improvement in serum IGF-1 with resistance exercise (two sets of 10 repetitions at 75% 1RM) is also present in older adults with MCI, though these changes in IGF-1 were not associated with improvements in cognition (Tsai et al., 2018). The vast majority of IGF-1 is secreted by the liver (Laron, 2001) with tissues like skeletal muscle also contributing to IGF-1 release during resistance exercise (Perrone et al., 1995). Despite the exercise-induced increase in IGF-1, this increase has not been shown to be associated improvements in executive function in young (Tsai et al., 2014) or older adults with MCI (Tsai et al., 2018). The absence of a relationship between increases in IGF-1 and improvements in cognition following resistance exercise may be attributed to the transient nature of the circulating IGF-1 response, as levels remain elevated for only ∼20 min (Tsai et al., 2014). IGF-1 is tonically regulated and in excess quantities is taken up by CSF. Therefore, a potential reason for the rapid return to baseline of IGF-1 post-exercise could be uptake into the brain via CSF (Pulford and Ishii, 2001). Despite the transient nature of resistance exercise-induced IGF-1 release, there is some evidence suggesting RT interventions may influence basal levels of IGF-1. The same work by Özbeyli et al mentioned in previous sections also demonstrated that 6 weeks of ladder-climbing RT improved basal serum IGF-1 levels in a rodent model of Alzheimer’s disease (Özbeyli et al., 2017). In humans, 2 years of progressive RT (full body RT; 2 sets of 2-8 repetitions with progression from 30%–55% 1RM, 2 times per week) increased basal levels of plasma IGF-1 in older women, with changes in IGF-1 concentration being associated with improvements in global cognition (Molina-Sotomayor et al., 2020). In contrast, 8 weeks of lower-limb RT (Walsh et al; exercise parameters highlighted in BDNF section) did not change basal serum IGF-1 (Walsh et al., 2015). Thus, it is possible that longer-term interventions are needed to elicit a change in basal serum IGF-1 concentrations in humans. Collectively, there is limited evidence to demonstrate that chronic RT may be improving cognitive function and protecting brain health through augmenting circulating IGF-1. The inconsistent results regarding IGF-1 responses to RT further suggests that the relationship between exercise and molecular factors underlying the neurocognitive benefits of exercise are complex and extend beyond IGF-1 and BDNF alone. Additional research is needed to clarify our current understanding, especially considering circulating IGF-1 responses to acute resistance exercise and RT have been understudied relative to BDNF.

### Effects of resistance exercise training on cerebrovascular health and function

Our group has previously reviewed the effects of habitual RT on cerebrovascular function across the lifespan (Allison and Al-Khazraji, 2024b). Habitual RT is associated with improved endothelial function (Silva et al., 2021), nitric oxide bioavailability (Güzel et al., 2007), resting arterial diameter (Zoeller et al., 2009), improved cerebral autoregulatory function (Roy et al., 2022), and importantly improved global (Xu et al., 2014) and regional CBF (Macaulay et al., 2022). Despite the clear associations between habitual participation in RT and improved vascular and cerebrovascular function, the mechanisms as they relate to neurodegenerative disease such as ADRD have not been discussed. Increased oxidative stress and inflammation ultimately disrupts proper endothelial function (Gryglewski et al., 1986), leading to impaired BBB integrity and cerebrovascular dysfunction (Krizbai et al., 2005), neurovascular coupling (Lourenço et al., 2017), and cerebrovascular reactivity (Mayhan et al., 2008). There are limited data related to effects of RT on cerebrovascular function in the elderly or in neurodegenerative conditions. Despite the paucity of data in older adults, findings from young healthy individuals may provide a fundamental backdrop for future study considerations. RT and its effects on arterial stiffness remain a topic of debate (Miyachi, 2013). Meta-analyses suggest that RT interventions increase aortic and carotid artery stiffness in healthy young men in an intensity dependent manner–such that higher intensities (75%–80% of 1RM) yield greater increases in central arterial stiffness (Kawano et al., 2006; Miyachi et al., 2004; Okamoto et al., 2009; Okamoto et al., 2006), but no such evidence has been observed in healthy young women (Morgan et al., 2023; Rossow et al., 2014), or older adults (Rossow et al., 2014; Cortez-Cooper et al., 2008; Ramírez-Vélez et al., 2020; Jefferson, 2014). Acutely, high intensity resistance exercise (80%–100% 1RM) has been reported to increase carotid artery stiffness for up to 30 min post-exercise (Lefferts et al., 2014), MCAv for at least 1-min post exercise, and MCA pulsatility index in the immediate (∼30s) post-exercise period (Koch et al., 2005). The increase in MCAv and MCA pulsatility index is likely a compensatory response to counter the drop in blood pressure following resistance exercise and maintain adequate cerebral perfusion (Lefferts et al., 2014; Koch et al., 2005). Indeed, the magnitude of post-exercise hypotension is commensurate with the pressure challenge during exercise (Chen and Bonham, 2010). On the surface, increased arterial stiffness in young men with habitual RT may raise concern due to its associations with hypertension (Safar, 2018), cardiovascular diseases (Mitchell et al., 2010), and cerebral microvascular damage (Badji et al., 2019; Tsao et al., 2013; Singer et al., 2014; Mitchell, 1985). However, the relationship between RT and arterial stiffness may need recontextualization and in our opinion should not be interpreted in the same lense as disease related arterial stiffening. There is evidence of chronic RT-induced adaptations supporting improved pulsatile dampening in the cerebral vasculature–in turn reducing pulsatile burden on the cerebral microvasculature in trained individuals (Nakamura et al., 2021). Perhaps the structural remodelling of arteries with RT is a compensatory adaptation to reduce arterial distension during high-grade resistance exercise-induced oscillations in blood pressure. Additional work is necessary to understand the broader cerebrovascular and neurological implications of RT-related arterial stiffening. Nonetheless, there is currently no evidence that RT is associated with negative effects on the cerebral vasculature despite the theoretical link between RT-induced arterial stiffness and microvascular damage.

Cross-sectionally, young healthy resistance-trained individuals (>6 months) have elevated cerebrovascular resistance and reduced cerebrovascular pulsatility during high intensity exercise. The cerebrovascular adaptations to RT are supported by work from Thomas et al, who conducted 12 weeks of progressive RT (progression from 60%–90% 1RM with repetitions lowering as intensity increases, 3 times per week) in healthy young adults and similarly observed increased cerebrovascular resistance and decreased pulsatility at rest. These findings are unique to RT, as Thomas et al observed no such changes in the age-matched aerobically trained group (Thomas et al., 2021). Further research is required to better understand cerebrovascular adaptations to RT, as increased resting cerebrovascular resistance is typically a sign of negative alterations in cerebrovascular function (Yew and Nation, 2017). As mentioned previously, increased cerebrovascular resistance at rest, whether it be related to aging or disease, is typically a negative change in cerebrovascular function and is a sign of greater flow impedance–ultimately leading to reductions in CBF (Claassen et al., 2021). In the work from Thomas et al, they observed a significant but relatively minor increase in resting mean arterial pressure (∼3 mmHg) and a reduction in resting MCAv (∼3 cm/s) after 12 weeks of RT. This is an important finding related to cerebrovascular function, as cerebrovascular resistance is an index measure calculated as mean arterial pressure divided by MCAv (Thomas et al., 2021). Therefore, even slight increases in resting mean arterial pressure alongside reductions in MCAv would lead to significant increases in cerebrovascular resistance. Elevated mean arterial pressure with RT reported in this study is of note due to the more frequently reported blood pressure lowering effects of RT reported across the literature, particularly in hypertensive individuals (Correia et al., 2023). Considering the positive effects of RT on blood pressure in older and hypertensive adults, it is unlikely that the ∼3 mmHg rise in resting blood pressure observed by Thomas et al is of clinical concern. Importantly, the authors of this work similarly did not interpret their findings related to cerebrovascular resistance as negative adaptations to RT, but instead consider the increase to be a protective response to reduce the effects of increased mean arterial pressure on the cerebral circulation - where hyperperfusion can damage the microvasculature (Thomas et al., 2021). Indeed, despite the increase in cerebrovascular resistance with 12 weeks of RT, the authors also observed a significant reduction in MCA pulsatility index–suggestive of improved pulsatile damping despite the increase in mean arterial pressure. Thus, in alignment with the interpretation from Thomas et al, perhaps changes in cerebrovascular resistance observed in RT are to be separated from vascular pathology and recontextualized as a favourable adaptation, as increased resistance may suppress transmission of pulsatile blood flow into the cerebral circulation during- and post-resistance exercise (Koch et al., 2005). Reduced cerebrovascular pulsatility with RT could also be interpreted as improved capacity for cerebral arteries to dampen pressure waves ejected from the heart. Nonetheless, more work is necessary to better understand the effects of RT on cerebrovascular structure and function, particularly in elderly and diseased populations.

As it relates to cerebral autoregulatory function, Koch et al demonstrated that acute resistance exercise (low load ∼20 repetitions at 50%–60% 1RM; high load ∼8 repetitions at 80%–90% 1RM) has been shown to significantly impair the cerebral autoregulation immediately post-exercise, demonstrating a stark increase in MCAv relative to blood pressure (autoregulatory gain) (Koch et al., 2005). Impaired cerebral autoregulation and the other metrics of cerebral hemodynamics reported by Koch et al (rapid rise in MCA pulsatility, drop in cerebrovascular resistance index) in the immediate post-exercise period align well with the phenomenon of *weightlifter’s blackout*–in which individuals may experience syncope or pre-syncope immediately following high intensity resistance exercise, particularly when in the upright standing position due to the sudden pressure drop upon release of the external load (Compton et al., 1973). However, the work from Koch et al is suggestive of a compensatory response to the sudden drop in blood pressure. Given that blood pressure drops dramatically following resistance exercise, an increase in pulsatility, a drop in resistance, and an overall increase in MCAv (and likely cerebral perfusion) are indicative of an emergency protective response to avoid pre-syncope and syncope (Koch et al., 2005). Thus, while metrics of cerebral autoregulation appear to be impaired immediately following resistance exercise, increased autoregulatory gain (change in MCAv relative to change in blood pressure) should not be misinterpreted as damaging but instead as oppositional to *weightlifter’s blackout*.

In untrained individuals, the cerebral circulation exhibits greater sensitivity to reductions (than increases) in mean arterial pressure, demonstrating a greater change in MCAv (and assumingly CBF) during a hypotensive stimulus compared to a hypertensive stimulus (Roy et al., 2022; Allison and Al-Khazraji, 2024b). Interestingly, based on cross-sectional studies, it appears that sensitivity to reductions and increases in mean arterial pressure are handled similarly by the cerebral vasculature in resistance-trained adults (Roy et al., 2022). Furthermore, recent work from Korad et al. suggests improved pressure-buffering capacity of the cerebral circulation in resistance-trained adults, in which trained participants exhibited a similar change in MCAv to controls during unilateral knee extension resistance exercise despite a greater systemic blood pressure response (Korad et al., 2024). While they did not investigate the directional sensitivity of the cerebral circulation in this study, their findings do support adaptation in the inherent directional sensitivity of the cerebral pressure-flow relationship. While longer-term interventional work is necessary to understand how RT directly influences directional sensitivity of the cerebral pressure-flow relationship, the findings from Korad et al and Roy et al do indeed suggest that frequent exposure to oscillatory pressure challenges via resistance exercise may habituate the arteries to better handle both elevations and drops in mean arterial pressure and avoid hyperperfusion and pre-syncopal and syncopal responses, respectively (Korad et al., 2024; Roy et al., 2022).

In cross-sectional work from Xu et al, higher global CBF was observed in older individuals who frequently participating in RT (Xu et al., 2014). One of the few training studies in older adults from Macaulay et al, reported increased regional CBF in the hippocampus, anterior cingulate, posterior cingulate, putamen, insula, occipital lobe, and temporal lobe following high-intensity (70%–85% 1RM) RT 3 times per week for 12 weeks (Macaulay et al., 2022). Though the effect size was small (attributed to short duration of the exercise intervention by authors), the ∼4% increase in temporal lobe CBF is of clinical interest due to the region’s relationship with memory and ADRD. Furthermore, Macaulay et al also observed significant increases in the fMRI-derived fraction of low frequency fluctuations in the cerebellum, right middle temporal gyrus, and bilateral inferior parietal lobes, representing greater functional neuronal integrity and connectivity between brain regions (Macaulay et al., 2022). Reduced fraction of low frequency fluctuations has been reported in MCI and ADRD (Yang et al., 2018), suggesting that RT may support regional neuronal activity and functional integrity. Considering the critical importance of CBF delivery in maintaining neuronal health and function, these findings alone warrant further interventional investigations on how long-term RT affects cerebrovascular function in older adults across the cognitive and neurodegenerative spectrum.

Many of the positive vascular and cerebrovascular adaptations to RT can be linked back to reduced ROS production, improved antioxidant defenses, and reduced inflammation. These adaptations underlie the improvements in endothelial function via habitual RT which are well established (Silva et al., 2021) and likely driving the beneficial effects on CBF and improved regulation of CBF with changes in arterial blood pressure that have been observed to date. However, the implications of these improvements on BBB permeability and function are an emerging area of study. Recent work from Cho et al in women aged 65–84 showed improvements in BBB integrity with 12 weeks of 3 times per week moderate intensity (10-14/20 RPE) resistance band-based RT. Importantly, alongside the observed improvement in BBB function was an enhancement of antioxidant capacity, increased vascular endothelial growth factor and BDNF signalling (Cho and Roh, 2022). The neurotrophic responses to RT alongside upregulation of endothelial growth factors, improved endothelial function, and reduced inflammation and oxidative damage together implicate a role of RT in restoration of BBB function. However, additional research is needed to establish protective effects of RT on the BBB.

### Effects of resistance exercise training on brain structure

Given the importance of brain structure as a predictive biomarker for neurological health (Ten Kate et al., 2018; Planche et al., 2019; Fein et al., 2000; Dicks et al., 2020), and its chronology in the neurodegenerative cascade (preceded by inflammation, oxidative stress, vascular dysregulation, and Aβ deposition) (Iturria-Medina et al., 2016), measures of brain structure can provide tangible insight into whether exercise as a therapeutic intervention is efficacious for slowing the rate of atrophy with age and neurodegenerative disease.

Most literature reports overall positive effects of RT on regional grey matter brain structure, but effects of RT on global brain structure are less clear. One study from Liu-Ambrose et al observed significant reductions in global brain volume following 1 year of high-intensity progressive RT (2 sets of 6-8 repetitions at 70%–85% of 1RM, either once or twice weekly) in older women free from MCI (Liu-Ambrose et al., 2010). Despite a reduction in whole brain volume with RT, there was a clear improvement in cognitive function following the RT intervention. The reduction in brain volume with RT has been observed in pharmaceutical trials for AD, and is hypothesized to be related to reduction in Aβ load (Fox et al., 2005). The authors use this rationale cautiously to reconcile the mismatch between improved cognition and reduced brain volume, stating Aβ clearance and subsequent shifts in cerebrospinal fluid may therefore have also partially explained their findings. Improvements in BBB function (in particular efflux of metabolic waste), and reduced Aβ burden have been reported with RT in animal models (Hashiguchi et al., 2020).

More research is necessary to elucidate underlying causes for reductions in brain volume in response to exercise in older adults, including specific measurement of Aβ and neurofibrillary tangles. In work from Best et al, there was no evidence of attenuated global grey matter atrophy with 1 year of high-intensity progressive RT (2 sets of 6-8 repetitions at 70%–85% of 1RM, either once or twice weekly) in 155 older women (Best et al., 2015). Though Best et al reported no change in grey matter atrophy rates, they did observe clear improvements in memory (Best et al., 2015). These findings suggest that positive effects of RT on brain function may occur without changes in total brain volume and grey matter volume.

Increases in global brain volume in response to exercise is not well supported; however, there is support that while exercise may not reverse age-related cortical decline, it does appear to reduce the rate of cortical decline compared to normal aging (Colcombe et al., 2003). Additionally, while the changes in global brain structure may be negligible, evidence does suggest that certain brain regions are particularly responsive to exercise effects. In cardiac rehab settings, 3 days per week of moderate-intensity aerobic exercise for a total of 6 months was associated with improved regional grey matter volume in the superior frontal gyrus, superior temporal gyrus, posterior cerebellum, and supplementary motor area (Anazodo et al., 2013). While these findings were in response to aerobic exercise training, they support the notion that not all regions of the brain are structurally responsive to the benefits of exercise. As it relates to RT interventions, importantly, the hippocampus and related subfields appear to be highly responsive to RT interventions (Broadhouse et al., 2020; Feter et al., 2018). Broadhouse et al demonstrated a clear protection of the left hippocampus with 6 months of high-intensity RT (3 sets of 8 repetitions at 80% 1RM, two or three times per week) compared to sham–an effect that persisted 1-year post-intervention in the 200 older adults studied (Broadhouse et al., 2020). In particular, there was a clear neuroprotective effect on the left CA1, dentate gyrus, and subiculum regions of the hippocampus, all showing a reduced atrophy rate compared to sham controls (Broadhouse et al., 2020). The CA1, dentate gyrus, and subiculum regions are of particular interest in the context of ADRD, as they are associated with autobiographical memory (Bartsch et al., 2011), encoding and retrieval of episodic memories (Hainmueller and Bartos, 2020), and hippocampal synaptic relay (Baset and Fengwen, 2024), respectively. Protection of these hippocampal subfields is critical in maintaining memory function with advanced age.

The benefits of RT on hippocampal subfields are further supported by recent work from Vints et al, demonstrating that even 12 weeks of progressive RT peaking at 85% of 1RM was sufficient in improving CA1, CA4, subiculum, and dentate gyrus volume in 70 older men and women (Vints et al., 2024). In contrast, a study investigating the effects of 6-month of either high intensity aerobic training or lower limb progressive RT (3 sets of 6–10 repetitions with intensity progression from 70%–85% 1RM, 2 times per week) on hippocampal volumes in 29 older women with probable MCI demonstrated a 5.6% and 2.5% increase in the left and right hippocampus, respectively, in response to aerobic exercise training–but observed no such positive effects of RT on hippocampal volumes (ten Brinke et al., 2015). Considering the small sample size in the RT group (N = 8), further study is necessary to determine the effects of RT on hippocampal volumes. The responsiveness of the hippocampus and its subfields to exercise are well supported by molecular mechanisms–as these brain regions exhibit elevated levels of neurotrophic factors (IGF-1; BDNF expression) with RT (Berbert-Gomes et al., 2024). Taken alongside the reported increases in regional CBF discussed previously, increased expression of neurotrophic factors in response to RT may facilitate neurogenesis and synaptic plasticity in the hippocampus and its subfields.

The improved brain structure in older adults after RT may also be related to increased cerebral glucose metabolism. Hippocampal atrophy has been shown to be strongly linked to cerebral glucose hypometabolism in older adults with AD (Yamaguchi et al., 1997). Though there are limited studies investigating the effects of RT on cerebral glucose metabolism, studies looking at the effects of 16 weeks of combined exercise interventions (aerobic and RT) reported significant improvements in glucose metabolism in the sensorimotor cortex in 172 elderly adults (Shah et al., 2014). 12 weeks of moderate-intensity RT (3 sets of 10–13 repetitions at 45%–65% 1RM, 3 times per week) has also been shown to improve systemic glucose metabolism in older adults with metabolic syndrome (Zhou et al., 2022). Indeed, decreased insulin sensitivity and impaired glucose metabolism systemically are linked to the progression of neurodegenerative diseases (Farooqui et al., 2012). Though the direct effects of RT on cerebral glucose metabolism across the cognitive spectrum remain unclear, the mechanistic rationale is sufficient to warrant future investigations on mechanisms underlying improved brain structure with habitual RT.

RT interventions have also been demonstrated to improve measures of white matter integrity. One year of high-intensity RT (2 sets of 6-8 repetitions at 70%–85% of 1RM) twice per week in 54 older women (65–75 years) reduced white matter hyperintensity progression compared to balance and toning controls. However there was no difference between controls and individuals who did the RT protocol only once per week–indicating a training frequency threshold of 2 times per week on neuroprotective effects, in line with current physical activity guidelines (Bolandzadeh et al., 2015). In a separate research article from the same cohort and intervention, 1 year of RT was associated with reductions in white matter hyperintensity related disruptions in the sensorimotor network, and dorsal and ventral attention networks (Crockett et al., 2022). Similarly, 4-month of thrice-weekly RT (3 sets of 8–15 repetitions at 50%–80% of 1RM) improved white matter density in 37 frail older women (Bucci et al., 2023). The observed effects in this study were also observed with physical phenotype, as frail women who were born to lean and normal-weight mothers conferred greater RT-related white matter benefits compared to those born to obese mothers. The underlying cause of these observations are likely to be multi-factorial, receiving contributions from metabolic, inflammatory, and vascular factors. In a previously mentioned study primarily investigating regional changes in CBF in response to RT, a reversal of the progression of white matter lesion volume - indicative of improved white matter perfusion - was also reported following 12 weeks of RT (Macaulay et al., 2022). Best et al similarly reported positive effects of RT on white matter structure, demonstrating a reduced rate of white matter atrophy in individuals in the RT group compared to controls after the 1-year training intervention as well as 1 year post-intervention (Best et al., 2015). These findings also align well with the work from Broadhouse et al, in which improvements in brain structure are maintained up to 1-year after cessation of training (Broadhouse et al., 2020). It is unclear whether these benefits in the RT groups compared to controls are related to residual physiological effects of training or whether they are a result of habit-forming related to the 1-year exercise intervention. Future works should consider collecting questionnaire or physical fitness data to determine the underlying cause of the long-term benefits of RT.

### Effects of resistance exercise training on cognitive function

The effects of acute and habitual exercise of any type on cognitive function are well established, with multiple quality reviews highlighting the effectiveness of resistance exercise on improved cognition across multiple domains (Landrigan et al., 2020; Chang et al., 2012; Herold et al., 2019; Cheng et al., 2022; Coelho-Junior et al., 2022). Acutely, it appears that low-to-moderate resistance exercise is effective in improving global cognition, as well as multiple aspects of executive function, cognitive flexibility, and processing speed, with inconsistent findings as it relates to memory function (Loprinzi et al., 2018). For the purposes of this review, memory will be the primary domain of interest due to its intimate relationship with MCI and ADRD. Cognitive performance in subdomains of memory also track well with brain regions most vulnerable to age- and disease-related atrophy (Fjell et al., 2014). In contrast to the equivocal effects of resistance exercise, other studies show strong associations between acute bouts of aerobic exercise and improved memory performance (Cantelon and Giles, 2021). It is unclear whether the discrepant impacts of resistance versus aerobic exercise on memory-related tasks in humans results from activation of different physiological pathways, individual differences in response to exercise, or different cognitive testing approaches used across studies.

The literature widely reports benefits of RT on global cognition (Suo et al., 2016; Coelho-Júnior et al., 2020; Fiatarone Singh et al., 2014; Chupel et al., 2017; Mavros et al., 2017) and executive function (Nouchi et al., 2014; de Oliveira Silva et al., 2019; Helmes and Harris, 2017) in older adults across the neurocognitive spectrum (cognitively normal and MCI) but free from diagnosed ADRD. Though acute effects of resistance exercise on memory function are contentious at best, it is reasonable to assume habitual RT would be more efficacious in improving memory given the associations between muscular strength and both memory (Shaughnessy et al., 2020; Liao et al., 2024; Cai et al., 2023) and hippocampal volume (Kim YS. et al., 2017; Firth et al., 2020; Meysami et al., 2023). Greater muscle strength in older adults is inversely related to the development of sarcopenia and frailty (Veen et al., 2021). Maintaining muscle strength and mass is critical for promoting higher functional capacity and physical independence with aging (dos Santos et al., 2017). The positive adaptations observed in both global and regional measures of perfusion and brain structure to RT detailed in the sections above also support reported benefits of RT on memory function. A study investigating the effects of thrice-weekly moderate intensity resistance-band RT with self-directed intensity progression in over 200 community dwelling older adults demonstrated marked improvements in working memory (Lachman et al., 2006). Furthermore, the magnitude of cognitive benefits of RT were associated with the strength gains made over 6 months–suggesting a dose-response relationship between increased muscular strength and memory function (Lachman et al., 2006). A study from Mavros et al reported that muscle gains via RT (3 sets of 8 repetitions at 80%–90% 1RM, 2-3 times per week), but not improvements in aerobic fitness over 6-month were related to improved memory performance in 100 community dwelling adults diagnosed with MCI (Mavros et al., 2017). Additionally, a 1-year training study in 155 older women demonstrated that not only was a single day of high intensity RT (2 sets of 6-8 repetitions at 70%–85% of 1RM) per week sufficient to improve memory at the cessation of the study compared to pre-training, but these cognitive benefits persisted for 1 year after training compared to balance-and-toning controls (Best et al., 2015). The relationship between exercise intensity and neurocognitive effects of RT is supported by work from Feter et al, who showed improvements in cognition over 12-week of high velocity RT (1–3 sets of 8–10 repetitions at 40%–60% 1RM, 2 times per week) were statistically mediated by the improvements in lower body strength, power, and muscle thickness (Feter et al., 2023), though they did not find any effects of the RT intervention on memory-related domains. The null findings on memory reported by Feter et al may be related to exercise intensity–as increases in muscle mass are not necessarily intensity dependent (Mcleod et al., 2024), but evidence does support that intensity mediates exercise-related neurogenesis and neuroplasticity in interval and aerobic exercise models (Marko et al., 2022; Freitas et al., 2018; Hoffmann et al., 2016; Kovacevic et al., 2020; Murawska-Ciałowicz et al., 2021). Another large-scale trial (N = 1335) similarly found no clear benefits of low-to-moderate intensity RT (2 sets of 15 repetitions at 60% 1RM, 5 times per week) on memory function in 2 years of training, further suggesting the importance of intensity in maximizing pro-cognitive effects of RT (Komulainen et al., 2010). It is important to note that in an investigation from Cassilhas et al directly comparing the effects of different intensities of RT (2 sets of 8 repetitions at 50% 1RM vs. 80% 1RM, both conducted 3 times per week), there appeared to be no differences in cognitive benefits (Cassilhas et al., 2007). The underlying factors for absence of differences in the work from Cassilhas et al despite clear mechanistic rationale and evidence for intensity-mediated neurocognitive benefits of RT remains unclear and warrants further study. Nonetheless, the literature does suggest that moderate-to-high intensities of RT is associated with significant improvements on memory function (Best et al., 2015; Coelho-Júnior and Uchida, 2021; Marston et al., 2019).

The cognitive benefits of RT may also be related to the inherent complexity of resistance exercise itself. As mentioned previously, resistance exercise requires individuals to remember exercises, machine settings, and training protocols, synchronize movements between several muscle different groups, and recruit the appropriate amount of muscle fibres to meet exercise demands. Thus, resistance exercise may act as a cognitive stimulus in and of itself (Forte et al., 2013). Furthermore, dual-task exercises that combine RT with simultaneous cognitive tasks may provide additional challenges, potentially enhancing cognitive outcomes. This idea is supported by Baek et al, who reported improved cognitive performance in older adults with cognitive impairment that completed 6-week of dual-task resistance band-based RT (3 sets of 10 repetitions, 3 times per week), which involved cognitively stimulating tasks simultaneously during RT (Baek et al., 2024). Several studies have demonstrated that resistance exercise, across varying intensities, improves psychological wellbeing without measurable changes in neurocognitive performance from pre to post-intervention (Tsutsumi et al., 1997; Peig-Chiello et al., 1998). The absence of improvements in neurocognitive performance may be due to limitations in study duration or the specific cognitive domains assessed. However, qualitative analyses of participants’ experiences in RT programs highlight perceived improvements in psychological wellbeing, which suggest enhanced self-efficacy—a critical factor for long-term adherence (Dionigi, 2007).

## Practical considerations for resistance training in older adults at risk of ADRD

### Adherence, adoption, and feasibility of resistance training interventions

Adherence to exercise programs, particularly RT, is a multifactorial challenge influenced by biopsychosocial factors. Barriers to adherence can be broadly categorized under the concept of self-efficacy, which refers to an individual’s confidence in their ability to succeed in specific tasks, including both physical and psychological aspects. Additionally, social support plays a critical role in overcoming these barriers (Dionigi, 2007). Effective programs must address both the initial adoption of exercise and the sustained maintenance of participation over time. A study by Furlano and Nagamatsu demonstrated that longer-term RT (2 sets of 6-8 repetitions at 70%–85% of 1RM, 3 times per week for 26 weeks) is generally feasible for older adults at risk of type 2 diabetes, a population often experiencing deficits in cognitive functioning and at elevated risk for AD (Furlano and Nagamatsu, 2021). Although recruitment posed challenges, the study achieved high retention (100%) and adherence (85.5%) rates within the RT group. Notably, 90% of participants reported enjoying the program, with many citing social interaction and increased feelings of strength, mobility, and fitness as their favorite aspects. Key factors influencing adherence included program characteristics such as session timing and location, social connectivity, enjoyment, perceived safety, and delivery by trained healthcare professionals (Furlano and Nagamatsu, 2021). These findings underscore the critical role of socialization and perceived physical benefits in driving adherence, suggesting that similar strategies could be applied across diverse populations with chronic health conditions. However, recruitment challenges highlight potential barriers to scaling similar interventions, particularly in populations with varying degrees of health or cognitive impairment. These studies highlight the importance of self-efficacy, social interaction, and program characteristics in promoting adherence, aligning with biopsychosocial models of adherence that emphasize the interplay between individual motivations, environmental factors, and perceived outcomes (Dionigi, 2007).

Personalized exercise prescriptions that cater to the unique needs of older adults are essential. Individualized program design, including adjustments to intensity and duration, is crucial for optimizing outcomes. RT programs can be adapted to various settings, including community centers, homes, and online platforms, increasing accessibility for a diverse population. For example, online RT programs demonstrate increased accessibility with high adherence levels and improved psychological wellbeing. In addition to being accessible and feasible, RT is cost-effective compared to balance and toning exercises, offering significant health and cognitive benefits. For instance, Davis et al found that 6 months of RT (2 sets of 6-8 repetitions at 70%–85% of 1RM, 2 times per week) in older adults with MCI led to greater cognitive improvements compared to the balance and toning group (Davis et al., 2013). Moreover, resistance training classes were more cost-effective for achieving each unit of Stroop test performance compared to balance and toning classes. These findings suggest that RT is a feasible, affordable, and effective option for individuals of varying health statuses, potentially promoting greater adherence after initial adoption. It is important to consider the role of total training load on exercise adherence in older adults, as training overload in older adults may lead to dropout. This is of particular concern in interventions using a multimodal approach of both aerobic and resistance exercise. Current recommendations suggest ∼150 min of moderate intensity aerobic exercise per week alongside moderate intensity RT at least twice per week (Nelson et al., 2007). While multimodal exercise has potential to negatively influence adherence by increasing total training volume, studies have demonstrated that multimodal or circuit-based exercise protocols are not only feasible but well-tolerated in older adults. A single session each of aerobic and resistance exercise per week (two total exercise sessions) was shown to elicit significant improvements in muscular strength and aerobic fitness similar in magnitude to twice and thrice weekly over the course of the 16-week intervention–though this study did not include cognitive of brain related measures (Fisher et al., 2013). Furthermore, all three groups (1 aerobic/week, 1 RT/week; 2 aerobic/week, 2 RT/week; 3 aerobic/week, 3 RT/week) had adherence rates of >88% over the duration of the 16-week protocol (Fisher et al., 2013). These findings suggest that combination exercise, particularly in older adults may be effective without necessarily needing to increase exercise frequency. Indeed, other work has demonstrated that RT twice weekly has been shown to improve cognitive performance (Bolandzadeh et al., 2015), and taken alongside low-moderate intensity aerobic exercise such as walking (Lee et al., 2025), is likely to be well received by older adults - particularly if there is a social element integrated into the program (Dionigi, 2007). Despite the numerous factors mentioned above, further research is still needed to explore long-term adherence and its determinants. Strategies to improve adherence include clear communication, addressing motivation and self-efficacy, and fostering social interactions while balancing exercise load and physical fatigue (Dionigi, 2007; Furlano and Nagamatsu, 2021). A biopsychosocial approach to program design can better address diverse health statuses and contribute to sustained engagement to ensure that participating individuals experience the psychological and physiological benefits of RT.

### Additional considerations in resistance training approaches

The combined effects of protein supplementation and resistance training on muscle mass, strength, and cognition in older adults remain a complex and debated area of research. RT is a well-established intervention for mitigating age-related muscle loss and weakness, and as discussed earlier, has demonstrated potential for improving cognitive functioning. However, the additional benefits of protein supplementation are less definitive, with studies reporting mixed outcomes (Griffen et al., 2024; Amasene et al., 2019). Some research suggests a synergistic relationship between protein intake and resistance exercise, noting that protein ingestion close to the time of exercise may enhance specific domains of cognitive functioning, like executive function, processing speed, memory, and reaction time (Griffen et al., 2024). However, many studies involving older adults have failed to demonstrate consistent added benefits of protein supplementation, potentially due to variations in the protein dosage and RT protocol itself. Amasene et al found that 12 weeks of RT (2 sets of individualized repetitions, progression of intensity from 50%–75% 1RM, 2 times per week) alone increased physical function, with no additional benefit from leucine-enriched whey protein supplementation (Amasene et al., 2019). In this study, authors noted that the dosage of protein after RT sessions may have been inadequate and suggested that participants may have required higher supplementation levels. Griffen et al aimed to address insufficient protein supplementation by examining how increasing protein intake, in isolation and with RT, in older adults would impact cognitive outcomes (Griffen et al., 2024). Their study found that whey protein supplementation at approximately 1.6 g/kg/day resulted in statistically significant improvements in executive function compared to the control group and the RT-only group. However, this improvement appeared domain-specific, as no significant differences were observed in working memory. Interestingly, RT (3 sets of 8–12 repetitions progressing from 50%–80% 1RM, 2 times per week) alone did not improve cognitive function in this study, nor did it exhibit synergistic effects with protein supplementation (Griffen et al., 2024). In addition to an underpowered analysis for the synergistic effects of protein and RT, Griffen et al noted that after final progressions to 80% 1RM, their RT protocol involved a ∼10% higher intensity than those examined in previous studies, potentially influencing the cognitive outcomes (Griffen et al., 2024). These discrepancies underscore the mixed results regarding the effects of protein supplementation and its interaction with RT. Future research should aim to disentangle these inconsistencies by carefully examining the specific health outcomes being measured (e.g., physical function versus cognitive function), the type and dosage of protein used, individual differences in protein intake, characteristics of the target population, and the design of RT protocols. A more nuanced understanding of these variables will help clarify the potential benefits of combining protein supplementation with RT in older adults.

Innovative approaches are essential for enhancing RT programs for older adults by addressing diverse needs and barriers. Various modalities of RT such as elastic bands (Yoon et al., 2017) and isometric exercise training (Hess and Smart, 2017), as well as different RT set structures, have shown efficacy in improving levels of critical neuronal growth factors, cognitive and physical function, and vascular and mental health. Other RT prescription structures, such as drop-set RT (several sets to failure with descending loads with each consecutive set), have also shown significant improvements in BDNF and nerve growth factor levels in middle-aged Korean women with obesity (Bang, 2023). This highlights the potential of drop-set RT for targeting populations with specific metabolic or health challenges, though its high intensity and tempo may limit broader applicability. Compared to RT alone, which was shown to improve psychological wellbeing and physical functioning in older adults with cognitive impairment, dual-task exercises (exercise plus cognitive stimulation) were shown to also improve cognitive functioning (Baek et al., 2024). This suggests that a dual-task exercise, which requires both cognitive and physical effort, may be a more potent stimulus that allows older adults to maximize the benefits from RT.

Blood flow restriction exercise is also a promising avenue for improving functional capacity in older adults where exercise with high external loads may not be well tolerated (Törpel et al., 2018). RT with blood flow restriction is an ideal alternative to RT alone for older adults facing physical barriers as it is a safer and more efficient approach to elicit improvements in muscle strength and hypertrophy while minimizing the risk of injury (Ramos-Campo et al., 2018). Importantly, acute blood flow restriction exercise using an occlusive cuff in the upper or lower limbs has been shown to elicit similar improvements in cognitive flexibility compared to traditional RT despite working with significantly lower loads (30% of 1RM in blood flow restricted RT vs. 70% of 1RM in traditional RT) (Batman et al., 2024). In addition to its muscle and strength building qualities despite a lower relative load, blood flow restriction exercise with low loads has been shown to have no adverse effects on vascular function or blood flow in older adults (Lopes et al., 2022; Kim J. et al., 2017). Furthermore, recent meta-analyses of 12 randomized control trials demonstrated that blood flow restriction exercise is associated with improved arterial endothelial function (as assessed via brachial artery flow mediated dilation test) (Zhang et al., 2022). As mentioned in previous sections, strength gains have been associated with cognitive improvements in response to exercise interventions (Mavros et al., 2017; Lachman et al., 2006; Feter et al., 2023). While the direct neuroprotective effects of blood flow restriction exercise remain unclear (Törpel et al., 2018), exercise of any form are suggested to have positive effects on brain function. Blood flow restriction both increases the efficiency of RT (from a strength and hypertrophy perspective) while simultaneously lowering the barrier to entry, thus making it an exciting focus for future research with implications on exercise prescription in older adults with physical limitations.

Alternative approaches leveraging technology—such as online platforms, mobile apps, and wearable devices—can improve adherence and streamline program delivery (Coletta et al., 2024; Lee et al., 2024). However, accessibility issues, such as technological literacy and cost, may limit their reach in underserved populations. One solution could be community-based programs, which combine cognitive and physical training with health education lectures. Indeed, these programs have been shown to improve both physical and cognitive function among community-dwelling older adults (Nose et al., 2023). Emerging approaches like Electrical Muscle Stimulation (EMS) combined with RT may also offer additive benefits. Thapa et al demonstrated that EMS combined with RT enhanced strength, physical performance, and cognitive outcomes in middle-aged and older women compared to RT alone. While these findings highlight the potential of EMS as a complementary strategy, future research could explore its application in clinical populations with limited physical capacity, where traditional RT may be less feasible (Thapa et al., 2023). These innovations underscore the importance of tailoring RT programs to individual needs through holistic approaches that address physical, psychological, and social factors (Iuliano et al., 2015). Future research should investigate long-term outcomes, cost-effectiveness, and the comparative benefits of these methods relative to traditional RT. Additionally, exploring how these approaches can be integrated with other interventions, such as dietary strategies, may further optimize overall health outcomes (Iuliano et al., 2015).

### Safety of resistance training in older adults and clinical populations

Despite the benefits of RT on multiple physiological systems, particularly related to muscle mass and reducing the risk of frailty and loss of independence, as of 2018 only 19% of older adults report participating in muscle strengthening activities of any kind (Hyde et al., 2021). The hesitancy for participation in RT in older adults can be largely attributed to safety concerns–a topic that has been debated in recent years (Fragala et al., 2019). These concerns are a valid barrier to entry, as RT-naïve older adults may very well incur injury without proper program design (i.e., exercise intensity, volume), technique, and supervision (Fragala et al., 2019). Additionally, resistance exercise has traditionally been considered hazardous (Sagiv, 2009) for individuals with cardiovascular dysfunction due to exaggerated blood pressure responses. However, with appropriate exercise prescription beginning at lower intensities and coupled with incremental progression, these effects are of less concern (Fragala et al., 2019). Importantly older adults participating in RT should be coached on proper breathing techniques, specifically to avoid the Valsalva maneuver during exercise to reduce the likelihood of problematic increases in blood pressure. With coached breathing and appropriate supervision, RT at low-to-moderate intensities are unlikely to elicit Valsalva maneuver related spikes in blood pressure, as the Valsalva maneuver is only involuntary at intensities >80% of 1RM (MacDougall et al., 1992). To further ensure safety during RT in older adults, prescribing machine-based exercise early on in programming as opposed to free-weights is an ideal alternative that typically will protect the lower back by avoiding spinal loading and compensatory movements through the back, and allow for greater control and balance during exercise with external load (Feigenbaum and Pollock, 1999). Thus, for promotion of participation in RT in older adults, lower intensity, machine-based exercise led by qualified exercise professionals is critical for safe implementation of RT (Fragala et al., 2019). Ultimately, while safety concerns related to participation in RT are valid, when exercise is prescribed and monitored appropriately, the benefits of RT outweigh the risks and have potential to dramatically improve quality of life and independence in older adults (Levinger et al., 2007).

Similar to otherwise healthy older adults, individuals living with chronic health conditions stand to greatly benefit from participation in RT. The positive effects of RT are well-documented, improving glycemic control in healthy adults (Ashton et al., 2020) and type-2 diabetics (Ishiguro et al., 2016; Jansson et al., 2022), reducing resting and ambulatory blood pressure in individuals with hypertension (Correia et al., 2023), enhancing muscular strength and function in sarcopenia (Zhao et al., 2022) and osteoarthritis (Lim et al., 2024), and positively influencing vascular health in those with cardiovascular disease (Paluch et al., 2024; Fecchio et al., 2021). Despite its effectiveness, several key considerations must be addressed when prescribing RT to ensure safety, adherence, and efficacy.

Medical clearance from a physician and individualized risk assessment are essential by certified healthcare and exercise professionals (Liguori and American College of Sports Medicine, 2020). In individuals with diabetes, careful attention to glycemic control is necessary, including monitoring blood glucose levels before and after training sessions and timing exercise to avoid periods of peak insulin activity (KANALEY et al., 2022). In hypertensive individuals, while RT can lower resting blood pressure over time, acute hypertensive responses during high-intensity or isometric lifts warrant caution (Vermeer et al., 1997; de Souza Nery et al., 2010). Submaximal loads and avoidance of the Valsalva maneuver via controlled breathing are recommended (Williams et al., 2007). Indeed, while progressive overload remains foundational to resistance training, but initial intensities should typically be low to moderate (e.g., 40%–60% of 1RM) with gradual increases to minimize injury risk (Paluch et al., 2024). For individuals with cardiovascular disease or stroke, factors such as impaired autonomic regulation, cerebrovascular function, and potential motor or balance deficits need to be considered and monitored during exercise sessions (Billinger et al., 2014). As such, programming may need to begin with machine-based or seated exercises, with gradual progression as physical function improves. When tailored appropriately to the individual’s clinical status, capacity, and goals, RT serves as an important element alongside aerobic exercise for management of chronic disease.

### Female-specific considerations in administration of resistance exercise

The feasibility and practical considerations discussed above apply to both males and females, but it is also important to acknowledge sex-specific barriers to RT–particularly as it pertains to older females. While in recent years the dogma of RT being inherently “masculine” has fallen out of favour, older adults may still possess pre-conceived notions about the concept of weightlifting. Though participation in RT has increased in females in recent years (Kraemer et al., 2025), many older females still report hesitancy, citing a lack of education and beliefs that any heavy lifting should be avoided (Hurst et al., 2023). The pre-conceived notion that RT needs to be done with heavy external loads is damaging and plays into the stereotype that there is no clear access point for RT-naïve older adults, and older females in particular. A lack of education and resources, taken alongside a generally lower level of baseline strength in older females compared to males, may deter females from initiating participation in RT programs and further promote the age-related loss in muscle and physical independence that disproportionately affects older females (de Jong et al., 2023). Hurst et al reported that although awareness and understanding of RT is low in older females, there is open mindedness towards resistance exercise as a modality so long as adequate peer and healthcare professional support is provided (Hurst et al., 2023). While participation in RT in older adulthood is key for females to combat age-related muscle loss, adoption of RT in females in midlife is of critical importance. Promotion of RT to maintain and build muscular strength in midlife greatly reduces the onset and progression of sarcopenia in older age (Vaishya et al., 2024). The primary barrier for participation in RT in midlife cited by females is related to time constraints and shifting life transitions, which include taking on primary caregiver roles to not only children but potentially aging parents as well (Stimson et al., 2024). Providing education on short duration resistance exercise provides an accessible and feasible approach to facilitate participation and adherence to RT and greatly reduces risk of frailty in older adulthood. In summary, addressing misconceptions, improving access to female-focused programming, and promoting time-efficient, low-barrier approaches to RT are critical to improving participation in older females.

### Summary

The neuroprotective effects of RT are multi-factorial and have direct effects on ADRD pathophysiological cascades. Initial benefits of RT primarily begin at the molecular level, as even an acute bout of resistance exercise reduces inflammation and increases antioxidant defenses (Özbeyli et al., 2017; Feter et al., 2019; Schimidt et al., 2019; Rondão et al., 2022; Franzke et al., 2018; Heneka et al., 2015; Steensberg et al., 2002; Hashiguchi et al., 2020; Forti et al., 2014; Ostrowski et al., 1999; Cassatella et al., 1993; Liu et al., 2020; Vilela et al., 2017; Vints et al., 2024), promotes expression of neurotrophic factors such as IGF-1 (Özbeyli et al., 2017; Cassilhas et al., 2012; Vega et al., 2010; Tsai et al., 2014; Molina-Sotomayor et al., 2020) and BDNF (Berbert-Gomes et al., 2024; Jafarzadeh et al., 2021; Yarrow et al., 2010; Walsh et al., 2015; Marston et al., 2017; Coelho et al., 2012; Pereira et al., 2013; Vaughan et al., 2014) in brain regions vulnerable to neurodegeneration (i.e., hippocampus). While reduction in inflammation and ROS production may not be sufficient to reverse Aβ and neurofibrillary tangle deposition; there is reason to believe that the production and accumulation of neurotoxic compounds in the parenchyma is attenuated with habitual RT, potentially preventing further accumulation. Furthermore, habitual RT is associated with improvements in endothelial function (Silva et al., 2021), cerebral perfusion (Xu et al., 2014; Macaulay et al., 2022), and glucose uptake in the brain (Yamaguchi et al., 1997; Shah et al., 2014), resulting in a better matching between neuronal metabolic supply and demand. The improved endothelial function reported with RT may also have implications on BBB function and integrity. In both animal and human studies, RT interventions have been shown to improve BBB structure and function (Hashiguchi et al., 2020; Cho and Roh, 2022), and likely underlie findings related to Aβ clear-out with habitual RT. Given that neuronal atrophy is largely driven by cerebral hypoperfusion and hypometabolism, a restoration in CBF via exercise has been shown to reverse or attenuate the rate of white matter damage (Macaulay et al., 2022; Best et al., 2015; Broadhouse et al., 2020; Bolandzadeh et al., 2015; Crockett et al., 2022; Bucci et al., 2023) and improve or attenuate age-related reductions in grey matter volumes, particularly in the hippocampus and its related subfields (Vints et al., 2024; Broadhouse et al., 2020; Feter et al., 2018). Restoration of cerebral tissues, increased expression of neurotrophic factors promoting neuroplasticity and neurogenesis, and improved efflux of neurotoxic Aβ with RT are likely to drive the subsequent improvements in cognition observed across the literature in several domains, particularly memory function (Gabriel et al., 2006; Best et al., 2015; Lachman et al., 2006; Coelho-Júnior and Uchida, 2021; Marston et al., 2019).

Ultimately, the neuroprotective cascade via habitual participation in resistance exercise hold promise for reducing risk of onset and reducing the rate of progression of ADRD. RT programs for older adults are most effective when they address adoption, adherence, and feasibility concerns, by integrating dietary strategies and innovative approaches to enhance outcomes (Dionigi, 2007; Davis et al., 2013; Griffen et al., 2024; Amasene et al., 2019; Lee et al., 2024). Employing methods such as dual-task training, community-based programs, and technology-based delivery, can improve cognitive and physical health while ensuring long-term engagement and accessibility (Baek et al., 2024; Coletta et al., 2024; Lee et al., 2024; Nose et al., 2023). Investigators should continue to prioritize conducting well-controlled randomized control trials to gain greater insight into the effects of multiple types of RT, multi-modal training, and community-based RT programs on brain health outcomes across the lifespan in both healthy and clinical populations. There should also be a priority to investigate whether RT has differential effects on males and females as it relates to cerebrovascular and brain health–as sexual dimorphism in exercise-related reduction in ADRD risk remains poorly understood. Future research should also consider personalized interventions, include measures of psychological and behavioral factors, and examine the combined effects of exercise and dietary interventions in a more nuanced manner, alongside the role of vascular health.

## References

[B1] Ahmad M. A. Kareem O. Khushtar M. Akbar M. Haque M. R. Iqubal A. (2022). Neuroinflammation: a potential risk for dementia. Int. J. Mol. Sci. 23 (2), 616. 10.3390/ijms23020616 35054805 PMC8775769

[B2] Alavi Naini S. M. Soussi-Yanicostas N. (2015). Tau hyperphosphorylation and oxidative stress, a critical vicious circle in neurodegenerative tauopathies? Oxidative Med. Cell. Longev. 2015 (1), 151979. 10.1155/2015/151979 26576216 PMC4630413

[B3] Allison E. Y. Al-Khazraji B. K. (2024a). Association of arterial stiffness index and brain structure in the UK biobank: a 10-year retrospective analysis. Aging Dis. 15 (4), 1872–1884. 10.14336/AD.2023.0419 37307821 PMC11272205

[B4] Allison E. Y. Al-Khazraji B. K. (2024b). Cerebrovascular adaptations to habitual resistance exercise with aging. Am. J. Physiology-Heart Circulatory Physiology 326 (3), H772–85. 10.1152/ajpheart.00625.2023 38214906 PMC11221804

[B5] Alty J. Farrow M. Lawler K. (2020). Exercise and dementia prevention. Pract. Neurol. 20 (3), 234–40. 10.1136/practneurol-2019-002335 31964800

[B6] Alwatban M. Murman D. L. Bashford G. (2019). Cerebrovascular reactivity impairment in preclinical alzheimer’s disease. J. Neuroimaging 29 (4), 493–8. 10.1111/jon.12606 30748053

[B7] Alwatban M. R. Aaron S. E. Kaufman C. S. Barnes J. N. Brassard P. Ward J. L. (2021). Effects of age and sex on middle cerebral artery blood velocity and flow pulsatility index across the adult lifespan. J. Appl. Physiol. 130 (6), 1675–83. 10.1152/japplphysiol.00926.2020 33703940 PMC8285608

[B8] Alzheimer’s Association (2019). Alzheimer’s disease facts and figures. Alzheimer’s and dementia 15 (3), 321–87. 10.1016/j.jalz.2019.01.010

[B9] Amasene M. Besga A. Echeverria I. Urquiza M. Ruiz J. R. Rodriguez-Larrad A. (2019). Effects of leucine-enriched whey protein supplementation on physical function in post-hospitalized older adults participating in 12-Weeks of resistance training program: a randomized controlled trial. Nutrients 11 (10), 2337. 10.3390/nu11102337 31581591 PMC6835698

[B10] Anazodo U. C. Shoemaker J. K. Suskin N. St. Lawrence K. S. (2013). An investigation of changes in regional gray matter volume in cardiovascular disease patients, pre and post cardiovascular rehabilitation. NeuroImage Clin. 3, 388–95. 10.1016/j.nicl.2013.09.011 24273722 PMC3814972

[B11] Anderson M. F. Åberg M. A. I. Nilsson M. Eriksson P. S. (2002). Insulin-like growth factor-I and neurogenesis in the adult mammalian brain. Dev. Brain Res. 134 (1–2), 115–22. 10.1016/s0165-3806(02)00277-8 11947942

[B12] Ashton R. E. Tew G. A. Aning J. J. Gilbert S. E. Lewis L. Saxton J. M. (2020). Effects of short-term, medium-term and long-term resistance exercise training on cardiometabolic health outcomes in adults: systematic review with meta-analysis. Br. J. sports Med. 54 (6), 341–8. 10.1136/bjsports-2017-098970 29934430

[B13] Badji A. Sabra D. Bherer L. Cohen-Adad J. Girouard H. Gauthier C. J. (2019). Arterial stiffness and brain integrity: a review of MRI findings. Ageing Res. Rev. 53, 100907. 10.1016/j.arr.2019.05.001 31063866

[B14] Baek J. E. Hyeon S. J. Kim M. Cho H. young Hahm S. C. (2024). Effects of dual-task resistance exercise on cognition, mood, depression, functional fitness, and activities of daily living in older adults with cognitive impairment: a single-blinded, randomized controlled trial. BMC Geriatr. 24 (1), 369. 10.1186/s12877-024-04942-1 38658827 PMC11044356

[B15] Baker L. D. Frank L. L. Foster-Schubert K. Green P. S. Wilkinson C. W. McTiernan A. (2010). Aerobic exercise improves cognition for older adults with glucose intolerance, a risk factor for Alzheimer’s disease. J. Alzheimer’s Dis. 22 (2), 569–79. 10.3233/JAD-2010-100768 20847403 PMC3049111

[B16] Bang H. S. (2023). Effect of resistance training with different set structures on neurotrophic factors and obesity-related biomarkers in middle-aged Korean women with obesity. J. Clin. Med. 12 (9), 3135. 10.3390/jcm12093135 37176576 PMC10179607

[B17] Bartsch T. Döhring J. Rohr A. Jansen O. Deuschl G. (2011). CA1 neurons in the human hippocampus are critical for autobiographical memory, mental time travel, and autonoetic consciousness. Proc. Natl. Acad. Sci. 108 (42), 17562–7. 10.1073/pnas.1110266108 21987814 PMC3198338

[B18] Baset A. Fengwen H. (2024). Shedding light on subiculum’s role in human brain disorders. Brain Res. Bull. 214, 110993. 10.1016/j.brainresbull.2024.110993 38825254

[B19] Batman G. B. Cooper C. B. Traylor M. K. Ransom K. V. Hill E. C. Hill B. D. (2024). Various modalities of resistance exercise promote similar acute cognitive improvements and hemodynamic increases in young, healthy adults. Cereb. Circulation - Cognition Behav. 7, 100363. 10.1016/j.cccb.2024.100363 39252851 PMC11381452

[B20] Berbert-Gomes C. Ramos J. S. Silveira-Rodrigues J. G. Leite D. M. M. Melo B. P. Soares D. D. (2024). An acute bout of resistance exercise increases BDNF in hippocampus and restores the long-term memory of insulin-resistant rats. Exp. Brain Res. 242 (4), 901–12. 10.1007/s00221-024-06795-x 38453752

[B21] Best J. R. Chiu B. K. Liang Hsu C. Nagamatsu L. S. Liu-Ambrose T. (2015). Long-Term effects of resistance exercise training on cognition and brain volume in older women: results from a randomized controlled trial. J. Int. Neuropsychological Soc. 21 (10), 745–56. 10.1017/S1355617715000673 26581787

[B22] Billinger S. A. Arena R. Bernhardt J. Eng J. J. Franklin B. A. Johnson C. M. (2014). Physical activity and exercise recommendations for stroke survivors: a statement for healthcare professionals from the American Heart association/American stroke association. Stroke 45 (8), 2532–53. 10.1161/STR.0000000000000022 24846875

[B23] Bliss E. S. Wong R. H. X. Howe P. R. C. Mills D. E. (2021). Benefits of exercise training on cerebrovascular and cognitive function in ageing. J. Cereb. Blood Flow. Metab. 41 (3), 447–70. 10.1177/0271678X20957807 32954902 PMC7907999

[B24] Bolandzadeh N. Tam R. Handy T. C. Nagamatsu L. S. Hsu C. L. Davis J. C. (2015). Resistance training and white matter lesion progression in older women: exploratory analysis of a 12-Month randomized controlled trial. J. Am. Geriatrics Soc. 63 (10), 2052–60. 10.1111/jgs.13644 26456233

[B25] Bolduc V. Thorin-Trescases N. Thorin E. (2013). Endothelium-dependent control of cerebrovascular functions through age: exercise for healthy cerebrovascular aging. Am. J. Physiology-Heart Circulatory Physiology 305 (5), H620–33. 10.1152/ajpheart.00624.2012 23792680

[B26] Bown C. W. Khan O. A. Moore E. E. Liu D. Pechman K. R. Cambronero F. E. (2021). Elevated aortic pulse wave velocity relates to longitudinal gray and white matter changes. Arteriosclerosis, Thrombosis, Vasc. Biol. 41 (12), 3015–24. 10.1161/ATVBAHA.121.316477 34706559 PMC8627676

[B27] Boyle P. A. Buchman A. S. Wilson R. S. Leurgans S. E. Bennett D. A. (2009). Association of muscle strength with the risk of alzheimer disease and the rate of cognitive decline in community-dwelling older persons. Archives Neurology 66 (11), 1339–44. 10.1001/archneurol.2009.240 19901164 PMC2838435

[B28] Brain J. Greene L. Tang E. Y. H. Louise J. Salter A. Beach S. (2023). Cardiovascular disease, associated risk factors, and risk of dementia: an umbrella review of meta-analyses. Front. Epidemiol. 3, 1095236. 10.3389/fepid.2023.1095236 38455934 PMC10910908

[B29] Broadhouse K. M. Singh M. F. Suo C. Gates N. Wen W. Brodaty H. (2020). Hippocampal plasticity underpins long-term cognitive gains from resistance exercise in MCI. NeuroImage Clin. 25, 102182. 10.1016/j.nicl.2020.102182 31978826 PMC6974789

[B30] Bucci M. Iozzo P. Merisaari H. Huovinen V. Lipponen H. Räikkönen K. (2023). Resistance training increases white matter density in frail elderly women. J. Clin. Med. 12 (7), 2684. 10.3390/jcm12072684 37048767 PMC10094827

[B31] Cai Z. Wang X. Wang Q. (2023). Does muscle strength predict working memory? A cross-sectional fNIRS study in older adults. Front. Aging Neurosci. 15, 1243283. 10.3389/fnagi.2023.1243283 37876877 PMC10590893

[B32] Calles-Escandon J. Cipolla M. (2001). Diabetes and endothelial dysfunction: a clinical perspective. Endocr. Rev. 22 (1), 36–52. 10.1210/edrv.22.1.0417 11159815

[B33] Campos H. C. Ribeiro D. E. Hashiguchi D. Glaser T. Milanis M. da S. Gimenes C. (2023). Neuroprotective effects of resistance physical exercise on the APP/PS1 mouse model of Alzheimer’s disease. Front. Neurosci., 17–2023. 10.3389/fnins.2023.1132825 37090809 PMC10116002

[B34] Cantelon J. A. Giles G. E. (2021). A review of cognitive changes during acute aerobic exercise. Front. Psychol. 12, 653158. 10.3389/fpsyg.2021.653158 34975602 PMC8716584

[B35] Carey J. R. Bhatt E. Nagpal A. (2005). Neuroplasticity promoted by task complexity. Exerc. sport Sci. Rev. 33 (1), 24–31. 15640717

[B36] Carro E. Trejo J. L. Gomez-Isla T. LeRoith D. Torres-Aleman I. (2002). Serum insulin-like growth factor I regulates brain amyloid-β levels. Nat. Med. 8 (12), 1390–7. 10.1038/nm1202-793 12415260

[B37] Carroll T. J. Riek S. Carson R. G. (2001). Neural adaptations to resistance training: implications for movement control. Sports Med. 31, 829–40. 10.2165/00007256-200131120-00001 11665911

[B38] Cassatella M. A. Meda L. Bonora S. Ceska M. Constantin G. (1993). Interleukin 10 (IL-10) inhibits the release of proinflammatory cytokines from human polymorphonuclear leukocytes. Evidence for an autocrine role of tumor necrosis factor and IL-1 beta in mediating the production of IL-8 triggered by lipopolysaccharide. J. Exp. Med. 178 (6), 2207–11. 10.1084/jem.178.6.2207 8245792 PMC2191270

[B39] Cassilhas R. C. Viana V. A. Grassmann V. Santos R. T. Santos R. F. Tufik S. (2007). The impact of resistance exercise on the cognitive function of the elderly. Med. and Sci. Sports and Exerc. 39 (8), 1401–7. 10.1249/mss.0b013e318060111f 17762374

[B40] Cassilhas R. C. Lee K. S. Fernandes J. Oliveira M. G. M. de Tufik S. Meeusen R. (2012). Spatial memory is improved by aerobic and resistance exercise through divergent molecular mechanisms. Neuroscience 202, 309–17. 10.1016/j.neuroscience.2011.11.029 22155655

[B41] Chang Y. K. Pan C. Y. Chen F. T. Tsai C. L. Huang C. C. (2012). Effect of resistance-exercise training on cognitive function in healthy older adults: a review. J. Aging Phys. Activity 20 (4), 497–517. 10.1123/japa.20.4.497 22186664

[B42] Cheignon C. Tomas M. Bonnefont-Rousselot D. Faller P. Hureau C. Collin F. (2018). Oxidative stress and the amyloid beta peptide in Alzheimer’s disease. Redox Biol. 14, 450–64. 10.1016/j.redox.2017.10.014 29080524 PMC5680523

[B43] Chen C. Y. Bonham A. C. (2010). Postexercise hypotension: central mechanisms. Exerc. Sport Sci. Rev. 38 (3), 122–127. 10.1097/JES.0b013e3181e372b5 20577060 PMC2936915

[B44] Chen J. J. Rosas H. D. Salat D. H. (2011). Age-associated reductions in cerebral blood flow are independent from regional atrophy. NeuroImage. 55 (2), 468–78. 10.1016/j.neuroimage.2010.12.032 21167947 PMC3435846

[B45] Chen P. Shen Z. Wang Q. Zhang B. Zhuang Z. Lin J. (2021). Reduced cerebral glucose uptake in an Alzheimer’s rat model with glucose-weighted chemical exchange saturation transfer imaging. Front. Aging Neurosci. 13, 618690. 10.3389/fnagi.2021.618690 33815088 PMC8010663

[B46] Cheng A. Zhao Z. Liu H. Yang J. Luo J. (2022). The physiological mechanism and effect of resistance exercise on cognitive function in the elderly people. Front. public health 10 (101616579), 1013734. 10.3389/fpubh.2022.1013734 36483263 PMC9723356

[B47] Cho S. Y. Roh H. T. (2022). Effects of exercise training on neurotrophic factors and blood–brain barrier permeability in young-old and old-old women. Int. J. Environ. Res. Public Health 19 (24), 16896. 10.3390/ijerph192416896 36554777 PMC9778715

[B48] Chung C. P. Lee H. Y. Lin P. C. Wang P. N. (2017). Cerebral artery pulsatility is associated with cognitive impairment and predicts dementia in individuals with subjective memory decline or mild cognitive impairment. J. Alzheimer’s Dis. 60 (2), 625–32. 10.3233/JAD-170349 28826186

[B49] Chupel M. U. Direito F. Furtado G. E. Minuzzi L. G. Pedrosa F. M. Colado J. C. (2017). Strength training decreases inflammation and increases cognition and physical fitness in older women with cognitive impairment. Front. physiology 8, 377. 10.3389/fphys.2017.00377 28659812 PMC5467003

[B50] Church D. D. Hoffman J. R. Mangine G. T. Jajtner A. R. Townsend J. R. Beyer K. S. (2016). Comparison of high-intensity vs. high-volume resistance training on the BDNF response to exercise. J. Appl. Physiology 121 (1), 123–8. 10.1152/japplphysiol.00233.2016 27231312

[B51] Cirulli F. Berry A. Chiarotti F. Alleva E. (2004). Intrahippocampal administration of BDNF in adult rats affects short-term behavioral plasticity in the morris water maze and performance in the elevated plus-maze. Hippocampus 14 (7), 802–7. 10.1002/hipo.10220 15382250

[B52] Claassen J. A. Zhang R. (2011). Cerebral autoregulation in Alzheimer’s disease. J. Cereb. Blood Flow and Metabolism 31 (7), 1572–7. 10.1038/jcbfm.2011.69 21540872 PMC3137479

[B53] Claassen JAHR Thijssen D. H. J. Panerai R. B. Faraci F. M. (2021). Regulation of cerebral blood flow in humans: physiology and clinical implications of autoregulation. Physiol. Rev. 101 (4), 1487–559. 10.1152/physrev.00022.2020 33769101 PMC8576366

[B54] Coelho F. M. Pereira D. S. Lustosa L. P. Silva J. P. Dias J. M. D. Dias R. C. D. (2012). Physical therapy intervention (PTI) increases plasma brain-derived neurotrophic factor (BDNF) levels in non-frail and pre-frail elderly women. Archives gerontology geriatrics 54 (3), 415–20. 10.1016/j.archger.2011.05.014 21684022

[B55] Coelho-Júnior H. J. Uchida M. C. (2021). Effects of low-speed and high-speed resistance training programs on frailty status, physical performance, cognitive function, and blood pressure in prefrail and frail older adults. Front. Med. 8, 702436. 10.3389/fmed.2021.702436 34381802 PMC8350041

[B56] Coelho-Júnior H. J. Oliveira Gonçalves I. D. Sampaio R. A. Sampaio P. Y. Lusa Cadore E. Calvani R. (2020). Effects of combined resistance and power training on cognitive function in older women: a randomized controlled trial. Int. J. Environ. Res. Public Health 17 (10), 3435. 10.3390/ijerph17103435 32423126 PMC7277751

[B57] Coelho-Junior H. Marzetti E. Calvani R. Picca A. Arai H. Uchida M. (2022). Resistance training improves cognitive function in older adults with different cognitive status: a systematic review and meta-analysis. Aging and Ment. Health 26 (2), 213–24. 10.1080/13607863.2020.1857691 33325273

[B58] Colcombe S. J. Erickson K. I. Raz N. Webb A. G. Cohen N. J. McAuley E. (2003). Aerobic fitness reduces brain tissue loss in aging humans. Journals Gerontology Ser. A Biol. Sci. Med. Sci. 58 (2), M176–80. 10.1093/gerona/58.2.m176 12586857

[B59] Coletta G. Noguchi K. S. Beaudoin K. D. McQuarrie A. Tang A. Griffin M. (2024). A live online exercise program for older adults improves depression and life-space mobility: a mixed-methods pilot randomized controlled trial. PLOS ONE 19 (11), e0312992. 10.1371/journal.pone.0312992 39527532 PMC11554215

[B60] Compton D. Hill P. M. Sinclair J. D. (1973). WEIGHT-LIFTERS’ BLACKOUT. Lancet 302 (7840), 1234–7. 10.1016/s0140-6736(73)90974-4 4128562

[B61] Correia P. R. Pansani A. Machado F. Andrade M. da Silva A. C. Scorza F. A. (2010). Acute strength exercise and the involvement of small or large muscle mass on plasma brain-derived neurotrophic factor levels. Clinics 65 (11), 1123–6. 10.1590/s1807-59322010001100012 21243284 PMC2999707

[B62] Correia R. R. Veras A. S. C. Tebar W. R. Rufino J. C. Batista V. R. G. Teixeira G. R. (2023). Strength training for arterial hypertension treatment: a systematic review and meta-analysis of randomized clinical trials. Sci. Rep. 13 (1), 201. 10.1038/s41598-022-26583-3 36604479 PMC9814600

[B63] Cortez-Cooper M. Y. Anton M. M. Devan A. E. Neidre D. B. Cook J. N. Tanaka H. (2008). The effects of strength training on central arterial compliance in middle-aged and older adults. Eur. J. Cardiovasc Prev. Rehabil. 15 (2), 149–55. 10.1097/HJR.0b013e3282f02fe2 18391640

[B64] Crockett R. A. Hsu C. L. Dao E. Tam R. Eng J. J. Handy T. C. (2022). Weight for it: resistance training mitigates white matter hyperintensity-related disruption to functional networks in older females. J. Alzheimer’s Dis. 90 (2), 553–63. 10.3233/JAD-220142 36155502

[B65] Dalle S. Rossmeislova L. Koppo K. (2017). The role of inflammation in age-related sarcopenia. Front. physiology 8, 1045. 10.3389/fphys.2017.01045 29311975 PMC5733049

[B66] Davis J. C. Bryan S. Marra C. A. Sharma D. Chan A. Beattie B. L. (2013). An economic evaluation of resistance training and aerobic training *versus* balance and toning exercises in older adults with mild cognitive impairment. PLOS ONE 8 (5), e63031. 10.1371/journal.pone.0063031 23690976 PMC3653911

[B67] De Boer A. Gaillard P. (2006). Blood–brain barrier dysfunction and recovery. J. Neural Transm. 113, 455–62. 10.1007/s00702-005-0375-4 16550324

[B68] de Heus R. A. A. de Jong D. L. K. Sanders M. L. van Spijker G. J. Oudegeest-Sander M. H. Hopman M. T. (2018). Dynamic regulation of cerebral blood flow in patients with alzheimer disease. Hypertension 72 (1), 139–50. 10.1161/HYPERTENSIONAHA.118.10900 29844143

[B69] de Jong D. L. K. de Heus R. A. A. Rijpma A. Donders R. Olde Rikkert M. G. M. Günther M. (2019). Effects of nilvadipine on cerebral blood flow in patients with alzheimer disease. Hypertension 74 (2), 413–20. 10.1161/HYPERTENSIONAHA.119.12892 31203725

[B70] de Jong JCBC Attema B. J. van der Hoek M. D. Verschuren L. Caspers M. P. M. Kleemann R. (2023). Sex differences in skeletal muscle-aging trajectory: same processes, but with a different ranking. GeroScience 45 (4), 2367–86. 10.1007/s11357-023-00750-4 36820956 PMC10651666

[B71] de Montgolfier O. Pinçon A. Pouliot P. Gillis M. A. Bishop J. Sled J. G. (2019). High systolic blood pressure induces cerebral microvascular endothelial dysfunction, neurovascular unit damage, and cognitive decline in mice. Hypertension 73 (1), 217–28. 10.1161/HYPERTENSIONAHA.118.12048 30571552

[B72] de Oliveira Silva F. Ferreira J. V. Plácido J. Sant’Anna P. Araújo J. Marinho V. (2019). Three months of multimodal training contributes to mobility and executive function in elderly individuals with mild cognitive impairment, but not in those with Alzheimer’s disease: a randomized controlled trial. Maturitas 126, 28–33. 10.1016/j.maturitas.2019.04.217 31239114

[B73] De Sá Souza H. de Melo C. M. Piovezan R. D. Miranda R. E. Carneiro-Junior M. A. Silva B. M. (2022). Resistance training improves sleep and anti-inflammatory parameters in sarcopenic older adults: a randomized controlled trial. Int. J. Environ. Res. Public Health 19 (23), 16322. 10.3390/ijerph192316322 36498393 PMC9736460

[B74] de Sousa C. V. Sales M. M. Rosa T. S. Lewis J. E. de Andrade R. V. Simões H. G. (2017). The antioxidant effect of exercise: a systematic review and meta-analysis. Sports Med. 47, 277–93. 10.1007/s40279-016-0566-1 27260682

[B75] de Souza Nery S. Gomides R. S. Silva G. V. da de Moraes Forjaz C. L. Mion D. Tinucci T. (2010). Intra-Arterial blood pressure response in hypertensive subjects during Low- and high-intensity resistance exercise. Clinics 65 (3), 271–7. 10.1590/S1807-59322010000300006 20360917 PMC2845767

[B76] Desgranges B. Baron J. C. de la Sayette V. Petit-Taboué M. C. Benali K. Landeau B. (1998). The neural substrates of memory systems impairment in Alzheimer’s disease. A PET study of resting brain glucose utilization. Brain a J. neurology 121 (4), 611–31. 10.1093/brain/121.4.611 9577389

[B77] Dicks E. van der Flier W. M. Scheltens P. Barkhof F. Tijms B. M. Alzheimer's Disease Neuroimaging Initiative (2020). Single-subject gray matter networks predict future cortical atrophy in preclinical Alzheimer's disease. Neurobiol. Aging 94, 71–80. 10.1016/j.neurobiolaging.2020.05.008 32585492

[B78] Ding Q. Vaynman S. Akhavan M. Ying Z. Gomez-Pinilla F. (2006). Insulin-like growth factor I interfaces with brain-derived neurotrophic factor-mediated synaptic plasticity to modulate aspects of exercise-induced cognitive function. Neuroscience 140 (3), 823–33. 10.1016/j.neuroscience.2006.02.084 16650607

[B79] Dionigi R. (2007). Resistance training and older adults’ beliefs about psychological benefits: the importance of self-efficacy and social interaction. J. Sport Exerc. Psychol. 29 (6), 723–46. 10.1123/jsep.29.6.723 18089901

[B80] dos Santos L. Cyrino E. S. Antunes M. Santos D. A. Sardinha L. B. (2017). Sarcopenia and physical independence in older adults: the independent and synergic role of muscle mass and muscle function. J. Cachexia, Sarcopenia Muscle 8 (2), 245–50. 10.1002/jcsm.12160 27897417 PMC5377449

[B81] Dost F. S. Erken N. Ontan M. S. Ates Bulut E. Kaya D. Kocyigit S. E. (2023). Muscle strength seems to be related to the functional status and severity of dementia in older adults with Alzheimer’s disease. Curr. Aging Sci. 16 (1), 75–83. 10.2174/1573411018666220616114641 35726809

[B82] Erickson M. A. Banks W. A. (2013). Blood–brain barrier dysfunction as a cause and consequence of Alzheimer’s disease. J. Cereb. Blood Flow and Metabolism 33 (10), 1500–13. 10.1038/jcbfm.2013.135 23921899 PMC3790938

[B83] Erickson K. Kramer A. F. (2009). Aerobic exercise effects on cognitive and neural plasticity in older adults. Br. J. sports Med. 43 (1), 22–4. 10.1136/bjsm.2008.052498 18927158 PMC2853472

[B84] Erickson K. I. Voss M. W. Prakash R. S. Basak C. Szabo A. Chaddock L. (2011). Exercise training increases size of hippocampus and improves memory. Proc. Natl. Acad. Sci. 108 (7), 3017–22. 10.1073/pnas.1015950108 21282661 PMC3041121

[B85] Esler M. Rumantir M. Wiesner G. Kaye D. Hastings J. Lambert G. (2001). Sympathetic nervous system and insulin resistance: from obesity to diabetes. Am. J. Hypertens. 14 (S7), 304S-309S–309S. 10.1016/s0895-7061(01)02236-1 11721888

[B86] Esteban-Cornejo I. Ho F. K. Petermann-Rocha F. Lyall D. M. Martinez-Gomez D. Cabanas-Sánchez V. (2022). Handgrip strength and all-cause dementia incidence and mortality: findings from the UK Biobank prospective cohort study. J. Cachexia, Sarcopenia Muscle 13 (3), 1514–25. 10.1002/jcsm.12857 35445560 PMC9178163

[B87] Farooqui A. A. Farooqui T. Panza F. Frisardi V. (2012). Metabolic syndrome as a risk factor for neurological disorders. Cell. Mol. Life Sci. 69 (5), 741–62. 10.1007/s00018-011-0840-1 21997383 PMC11115054

[B88] Fecchio R. Y. Brito L. C. Pecanha T. de Moraes Forjaz C. L. (2021). Potential mechanisms behind the blood pressure–lowering effect of dynamic resistance training. Curr. Hypertens. Rep. 23 (6), 35. 10.1007/s11906-021-01154-5 34152491

[B89] Feigenbaum M. S. Pollock M. L. (1999). Prescription of resistance training for health and disease. Med. and Sci. Sports and Exerc. 31 (1), 38–45. 10.1097/00005768-199901000-00008 9927008

[B90] Fein G. Di Sclafani V. Tanabe J. Cardenas V. Weiner M. Jagust W. (2000). Hippocampal and cortical atrophy predict dementia in subcortical ischemic vascular disease. Neurology 55 (11), 1626–35. 10.1212/wnl.55.11.1626 11113215 PMC2733356

[B91] Ferrari B. L. Neto G. de C. C. Nucci M. P. Mamani J. B. Lacerda S. S. Felício A. C. (2019). The accuracy of hippocampal volumetry and glucose metabolism for the diagnosis of patients with suspected Alzheimer’s disease, using automatic quantitative clinical tools. Medicine 98 (45), e17824. 10.1097/MD.0000000000017824 31702636 PMC6855664

[B92] Ferreira M. E. S. de Vasconcelos A. S. da Costa Vilhena T. da Silva T. L. da Silva Barbosa A. Gomes A. R. Q. (2015). Oxidative stress in Alzheimer’s disease: should we keep trying antioxidant therapies? Cell. Mol. Neurobiol. 35, 595–614. 10.1007/s10571-015-0157-y 25616523 PMC11486210

[B93] Feter N. Penny J. C. Freitas M. P. Rombaldi A. J. (2018). Effect of physical exercise on hippocampal volume in adults: systematic review and meta-analysis. Sci. and Sports 33 (6), 327–38. 10.1016/j.scispo.2018.02.011

[B94] Feter N. Spanevello R. M. Soares M. S. P. Spohr L. Pedra N. S. Bona N. P. (2019). How does physical activity and different models of exercise training affect oxidative parameters and memory? Physiology Behav. 201, 42–52. 10.1016/j.physbeh.2018.12.002 30552921

[B95] Feter N. Schaun G. Z. Smith E. C. Cassuriaga J. Alt R. Redig L. (2023). High-velocity resistance training improves executive function in mobility-limited older adults. Archives Gerontology Geriatrics 114, 105081. 10.1016/j.archger.2023.105081 37269697

[B96] Fiatarone Singh M. A. Gates N. Saigal N. Wilson G. C. Meiklejohn J. Brodaty H. (2014). The study of mental and resistance training (SMART) Study—resistance training And/Or cognitive training in mild cognitive impairment: a randomized, double-blind, double-sham controlled trial. J. Am. Med. Dir. Assoc. 15 (12), 873–80. 10.1016/j.jamda.2014.09.010 25444575

[B97] Filardi M. Barone R. Bramato G. Nigro S. Tafuri B. Frisullo M. E. (2022). The relationship between muscle strength and cognitive performance across Alzheimer’s disease clinical continuum. Front. Neurology 13, 833087. 10.3389/fneur.2022.833087 35645971 PMC9133788

[B98] Firth J. A. Smith L. Sarris J. Vancampfort D. Schuch F. Carvalho A. F. (2020). Handgrip strength is associated with hippocampal volume and white matter hyperintensities in major depression and healthy controls: a UK biobank study. Biopsychosoc. Sci. Med. 82 (1), 39–46. 10.1097/PSY.0000000000000753 31702599

[B99] Fisher G. McCarthy J. P. Zuckerman P. A. Bryan D. R. Bickel C. S. Hunter G. R. (2013). Frequency of combined resistance and aerobic training in older women. J. Strength and Cond. Res. 27 (7), 1868–1876. 10.1519/JSC.0b013e31827367e0 22996024 PMC4066209

[B100] Fisher-Wellman K. Bloomer R. J. (2009). Acute exercise and oxidative stress: a 30 year history. Dyn. Med. 8 (1), 1. 10.1186/1476-5918-8-1 19144121 PMC2642810

[B101] Fjell A. M. McEvoy L. Holland D. Dale A. M. Walhovd K. B. Alzheimer’s Disease Neuroimaging Initiative (2014). What is normal in normal aging? Effects of aging, amyloid and Alzheimer’s disease on the cerebral cortex and the hippocampus. Prog. Neurobiol. 117, 20–40. 10.1016/j.pneurobio.2014.02.004 24548606 PMC4343307

[B102] Forte R. Boreham C. A. Leite J. C. De Vito G. Brennan L. Gibney E. R. (2013). Enhancing cognitive functioning in the elderly: multicomponent vs resistance training. Clin. Interventions Aging 8 (null), 19–27. 10.2147/CIA.S36514 23341738 PMC3546758

[B103] Forti L. N. Njemini R. Beyer I. Eelbode E. Meeusen R. Mets T. (2014). Strength training reduces circulating interleukin-6 but not brain-derived neurotrophic factor in community-dwelling elderly individuals. Age 36 (5), 9704. 10.1007/s11357-014-9704-6 25128203 PMC4453935

[B104] Fox N. Black R. Gilman S. Rossor M. Griffith S. Jenkins L. (2005). Effects of abeta immunization (AN1792) on MRI measures of cerebral volume in alzheimer disease. Neurology 64 (9), 1563–72. 10.1212/01.WNL.0000159743.08996.99 15883317

[B105] Fragala M. S. Cadore E. L. Dorgo S. Izquierdo M. Kraemer W. J. Peterson M. D. (2019). Resistance training for older adults: position statement from the national strength and conditioning association. J. Strength and Cond. Res. 33 (8), 2019–2052. 10.1519/JSC.0000000000003230 31343601

[B106] Franzke B. Schober-Halper B. Hofmann M. Oesen S. Tosevska A. Henriksen T. (2018). Age and the effect of exercise, nutrition and cognitive training on oxidative stress – the vienna active aging study (VAAS), a randomized controlled trial. Free Radic. Biol. Med. 121, 69–77. 10.1016/j.freeradbiomed.2018.04.565 29698742

[B107] Freitas D. A. Soares B. A. Nonato L. F. Fonseca S. R. Martins J. B. Mendonça V. A. (2018). High intensity interval training modulates hippocampal oxidative stress, BDNF and inflammatory mediators in rats. Physiology and Behav. 184, 6–11. 10.1016/j.physbeh.2017.10.027 29111230

[B108] Frith E. Loprinzi P. D. (2018). The association between lower extremity muscular strength and cognitive function in a national sample of older adults. J. lifestyle Med. 8 (2), 99–104. 10.15280/jlm.2018.8.2.99 30474005 PMC6239135

[B109] Furlano J. A. Nagamatsu L. S. (2021). Feasibility of a 26-Week exercise program to improve brain health in older adults at risk for type 2 diabetes: a pilot study. Can. J. Diabetes 45 (6), 546–52. 10.1016/j.jcjd.2020.11.001 33358932

[B110] Gabriel D. A. Kamen G. Frost G. (2006). Neural adaptations to resistive exercise: mechanisms and recommendations for training practices. Sports Med. 36, 133–49. 10.2165/00007256-200636020-00004 16464122

[B111] Gao Y. Z. Zhang J. J. Liu H. Wu G. Y. Xiong L. Shu M. (2013). Regional cerebral blood flow and cerebrovascular reactivity in Alzheimer’s disease and vascular dementia assessed by arterial spinlabeling magnetic resonance imaging. Curr. neurovascular Res. 10 (1), 49–53. 10.2174/156720213804806016 23151075

[B112] Gleeson M. Bishop N. C. Stensel D. J. Lindley M. R. Mastana S. S. Nimmo M. A. (2011). The anti-inflammatory effects of exercise: mechanisms and implications for the prevention and treatment of disease. Nat. Rev. Immunol. 11 (9), 607–15. 10.1038/nri3041 21818123

[B113] Glodzik L. Randall C. Rusinek H. de Leon M. J. (2013). Cerebrovascular reactivity to carbon dioxide in Alzheimer’s disease. J. Alzheimer’s Dis. 35 (3), 427–40. 10.3233/JAD-122011 23478306 PMC3776495

[B114] Goekint M. De Pauw K. Roelands B. Njemini R. Bautmans I. Mets T. (2010). Strength training does not influence serum brain-derived neurotrophic factor. Eur. J. Appl. physiology 110, 285–93. 10.1007/s00421-010-1461-3 20467874

[B115] Gratas-Delamarche A. Derbré F. Vincent S. Cillard J. (2014). Physical inactivity, insulin resistance, and the oxidative-inflammatory loop. Free Radic. Res. 48 (1), 93–108. 10.3109/10715762.2013.847528 24060092

[B116] Griffen C. Cullen T. Hattersley J. Weickert M. O. Dallaway A. Duncan M. (2024). Effects of resistance exercise and whey protein supplementation on cognitive function in older men: secondary analysis of a randomised, double-blind, placebo-controlled trial. Exp. Gerontol. 193, 112477. 10.1016/j.exger.2024.112477 38844183

[B117] Gryglewski R. Palmer R. Moncada S. (1986). Superoxide anion is involved in the breakdown of endothelium-derived vascular relaxing factor. Nature 320 (6061), 454–6. 10.1038/320454a0 3007998

[B118] Guo C. Sun L. Chen X. Zhang D. (2013). Oxidative stress, mitochondrial damage and neurodegenerative diseases. Neural Regen. Res. 8 (21), 2003–14. 10.3969/j.issn.1673-5374.2013.21.009 25206509 PMC4145906

[B119] Güzel N. A. Hazar S. Erbas D. (2007). Effects of different resistance exercise protocols on nitric oxide, lipid peroxidation and creatine kinase activity in sedentary males. J. sports Sci. and Med. 6 (4), 417–422. 24149472 PMC3794479

[B120] Habes M. Grothe M. J. Tunc B. McMillan C. Wolk D. A. Davatzikos C. (2020). Disentangling heterogeneity in alzheimer’s disease and related dementias using data-driven methods. Biol. Psychiatry 88 (1), 70–82. 10.1016/j.biopsych.2020.01.016 32201044 PMC7305953

[B121] Hainmueller T. Bartos M. (2020). Dentate gyrus circuits for encoding, retrieval and discrimination of episodic memories. Nat. Rev. Neurosci. 21 (3), 153–68. 10.1038/s41583-019-0260-z 32042144 PMC7115869

[B122] Hardy J. A. Higgins G. A. (1992). Alzheimer’s disease: the amyloid Cascade hypothesis. Science 256 (5054), 184–5. 10.1126/science.1566067 1566067

[B123] Hashiguchi D. Campos H. C. Wuo-Silva R. Faber J. Gomes da Silva S. Coppi A. A. (2020). Resistance exercise decreases amyloid load and modulates inflammatory responses in the APP/PS1 mouse model for Alzheimer’s disease. J. Alzheimer’s Dis. 73 (4), 1525–39. 10.3233/JAD-190729 31958083

[B124] Helmes E. Harris S. (2017). Exercise and executive functioning in older women. J. Women and Aging 29 (5), 376–84. 10.1080/08952841.2016.1256736 28027014

[B125] Heneka M. T. Carson M. J. El Khoury J. Landreth G. E. Brosseron F. Feinstein D. L. (2015). Neuroinflammation in Alzheimer’s disease. Lancet Neurology 14 (4), 388–405. 10.1016/S1474-4422(15)70016-5 25792098 PMC5909703

[B126] Herholz K. Salmon E. Perani D. Baron J. C. Holthoff V. Frölich L. (2002). Discrimination between alzheimer dementia and controls by automated analysis of multicenter FDG PET. Neuroimage. 17 (1), 302–16. 10.1006/nimg.2002.1208 12482085

[B127] Herold F. Törpel A. Schega L. Müller N. G. (2019). Functional and/or structural brain changes in response to resistance exercises and resistance training lead to cognitive improvements – a systematic review. Eur. Rev. Aging Phys. Activity 16 (1), 10. 10.1186/s11556-019-0217-2 31333805 PMC6617693

[B128] Hess N. C. L. Smart N. A. (2017). Isometric exercise training for managing vascular risk factors in mild cognitive impairment and alzheimer’s disease. Front. Aging Neurosci. 9, 48. 10.3389/fnagi.2017.00048 28316570 PMC5334511

[B129] Himali J. J. Baril A. A. Cavuoto M. G. Yiallourou S. Wiedner C. D. Himali D. (2023). Association between slow-wave sleep loss and incident dementia. JAMA neurol. 80 (12), 1326–33. 10.1001/jamaneurol.2023.3889 37902739 PMC10616771

[B130] Hoffmann K. Sobol N. A. Frederiksen K. S. Beyer N. Vogel A. Vestergaard K. (2016). Moderate-to-high intensity physical exercise in patients with Alzheimer’s disease: a randomized controlled trial. J. Alzheimer’s Dis. 50 (2), 443–53. 10.3233/JAD-150817 26682695

[B131] Hoiland R. L. Fisher J. A. Ainslie P. N. (2019). “Regulation of the cerebral circulation by arterial carbon dioxide,” in Comprehensive physiology (American Cancer Society), 1101–54. 10.1002/cphy.c180021 31187899

[B132] Holth J. K. Fritschi S. K. Wang C. Pedersen N. P. Cirrito J. R. Mahan T. E. (2019). The sleep-wake cycle regulates brain interstitial fluid tau in mice and CSF tau in humans. Science 363 (6429), 880–4. 10.1126/science.aav2546 30679382 PMC6410369

[B133] Hoyer S. Oesterreich K. Wagner O. (1988). Glucose metabolism as the site of the primary abnormality in early-onset dementia of Alzheimer type? J. neurology 235, 143–8. 10.1007/BF00314304 3367161

[B134] Huang W. Zhang X. Chen W. (2016). Role of oxidative stress in Alzheimer’s disease. Biomed. Rep. 4 (5), 519–22. 10.3892/br.2016.630 27123241 PMC4840676

[B135] Hurst C. Dismore L. Granic A. Tullo E. Noble J. M. Hillman S. J. (2023). Attitudes and barriers to resistance exercise training for older adults living with multiple long-term conditions, frailty, and a recent deterioration in health: qualitative findings from the Lifestyle in Later life – older People’s medicine (LiLL-OPM) study. BMC Geriatr. 23 (1), 772. 10.1186/s12877-023-04461-5 38001414 PMC10675908

[B136] Hyde E. T. Whitfield G. P. Omura J. D. Fulton J. E. Carlson S. A. (2021). Trends in meeting the physical activity guidelines: muscle-strengthening alone and combined with aerobic activity, United States, 1998–2018. J. Phys. Activity Health 18 (S1), S37–44. 10.1123/jpah.2021-0077 34465652 PMC11000248

[B137] Ionescu-Tucker A. Cotman C. W. (2021). Emerging roles of oxidative stress in brain aging and Alzheimer’s disease. Neurobiol. Aging 107, 86–95. 10.1016/j.neurobiolaging.2021.07.014 34416493

[B138] Ishiguro H. Kodama S. Horikawa C. Fujihara K. Hirose A. S. Hirasawa R. (2016). In search of the ideal resistance training program to improve glycemic control and its indication for patients with type 2 diabetes mellitus: a systematic review and meta-analysis. Sports Med. 46 (1), 67–77. 10.1007/s40279-015-0379-7 26604100

[B139] Iturria-Medina Y. Sotero R. C. Toussaint P. J. Mateos-Pérez J. M. Evans A. C. Weiner M. W. (2016). Early role of vascular dysregulation on late-onset alzheimer’s disease based on multifactorial data-driven analysis. Nat. Commun. 7 (1), 11934. 10.1038/ncomms11934 27327500 PMC4919512

[B140] Iuliano E. di Cagno A. Aquino G. Fiorilli G. Mignogna P. Calcagno G. (2015). Effects of different types of physical activity on the cognitive functions and attention in older people: a randomized controlled study. Exp. Gerontol. 70, 105–10. 10.1016/j.exger.2015.07.008 26183691

[B141] Jack C. R. Knopman D. S. Jagust W. J. Shaw L. M. Aisen P. S. Weiner M. W. (2010). Hypothetical model of dynamic biomarkers of the Alzheimer’s pathological cascade. Lancet Neurology 9 (1), 119–28. 10.1016/S1474-4422(09)70299-6 20083042 PMC2819840

[B142] Jafarzadeh G. Shakerian S. Farbood Y. Ghanbarzadeh M. (2021). Effects of eight weeks of resistance exercises on neurotrophins and trk receptors in alzheimer model Male wistar rats. Basic Clin. Neurosci. 12 (3), 349–359. 10.32598/bcn.2021.2067.1 34917294 PMC8666928

[B143] Jansson A. K. Chan Li X. Lubans D. R. Duncan M. J. Plotnikoff R. C. (2022). Effect of resistance training on HbA1c in adults with type 2 diabetes mellitus and the moderating effect of changes in muscular strength: a systematic review and meta-analysis. BMJ Open Diabetes Res. and Care 10 (2), e002595. 10.1136/bmjdrc-2021-002595 35273011 PMC8915309

[B144] Jay Widmer R. Lerman A. (2014). Endothelial dysfunction and cardiovascular disease. Vol. 2014, global cardiology science and practice. Doha, Qatar: Hamad bin Khalifa University Press HBKU Press. Available online at: https://www.qscience.com/ 10.5339/gcsp.2014.43 PMC4352682 25780786

[B145] Jefferson M. E. (2014). Abstract 404: the effects of resistance training and weight loss on arterial stiffness in older adults. Arteriosclerosis, Thrombosis, Vasc. Biol. 34 (Suppl. l_1), A404. 10.1161/atvb.34.suppl_1.404

[B146] Jefferson A. L. Cambronero F. E. Liu D. Moore E. E. Neal J. E. Terry J. G. (2018). Higher aortic stiffness is related to lower cerebral blood flow and preserved cerebrovascular reactivity in older adults. Circulation 138 (18), 1951–62. 10.1161/CIRCULATIONAHA.118.032410 30018169 PMC6394409

[B147] Jiang X. Lewis C. E. Allen N. B. Sidney S. Yaffe K. (2023). Premature cardiovascular disease and brain health in midlife: the CARDIA study. Neurology 100 (14), e1454–63. 10.1212/WNL.0000000000206825 36697246 PMC10104620

[B148] Kanaley J. A. Colberg S. R. Corcoran M. H. Malin S. K. Rodriguez N. R. Crespo C. J. (2022). Exercise/physical activity in individuals with type 2 diabetes: a consensus statement from the American college of sports medicine. Med. and Sci. Sports and Exerc. 54 (2), 353–368. 10.1249/MSS.0000000000002800 35029593 PMC8802999

[B149] Kang J. E. Lim M. M. Bateman R. J. Lee J. J. Smyth L. P. Cirrito J. R. (2009). Amyloid-β dynamics are regulated by orexin and the sleep-wake cycle. Science. 326 (5955), 1005–7. 10.1126/science.1180962 19779148 PMC2789838

[B150] Kawano H. Tanaka H. Miyachi M. (2006). Resistance training and arterial compliance: keeping the benefits while minimizing the stiffening. J. Hypertens. 24 (9), 1753–9. 10.1097/01.hjh.0000242399.60838.14 16915024

[B151] Kim Y. S. Shin S. K. Hong S. B. Kim H. J. (2017a). The effects of strength exercise on hippocampus volume and functional fitness of older women. Exp. Gerontol. 97, 22–8. 10.1016/j.exger.2017.07.007 28716635

[B152] Kim J. Lang J. A. Pilania N. Franke W. D. (2017b). Effects of blood flow restricted exercise training on muscular strength and blood flow in older adults. Exp. Gerontol. 99, 127–32. 10.1016/j.exger.2017.09.016 28986234

[B153] Kim D. Hughes T. M. Lipford M. E. Craft S. Baker L. D. Lockhart S. N. (2021). Relationship between cerebrovascular reactivity and cognition among people with risk of cognitive decline. Front. Physiology 12, 645342. 10.3389/fphys.2021.645342 34135768 PMC8201407

[B154] Kinney J. W. Bemiller S. M. Murtishaw A. S. Leisgang A. M. Salazar A. M. Lamb B. T. (2018). Inflammation as a central mechanism in Alzheimer’s disease. Alzheimer’s and Dementia Transl. Res. and Clin. Interventions 4, 575–90. 10.1016/j.trci.2018.06.014 30406177 PMC6214864

[B155] Klein A. B. Williamson R. Santini M. A. Clemmensen C. Ettrup A. Rios M. (2011). Blood BDNF concentrations reflect brain-tissue BDNF levels across species. Int. J. Neuropsychopharmacol. 14 (3), 347–53. 10.1017/S1461145710000738 20604989

[B156] Kleinloog J. P. D. Mensink R. P. Ivanov D. Adam J. J. Uludağ K. Joris P. J. (2019). Aerobic exercise training improves cerebral blood flow and executive function: a randomized, controlled cross-over trial in sedentary older men. Front. Aging Neurosci. 11, 333. 10.3389/fnagi.2019.00333 31866855 PMC6904365

[B157] Knopman D. S. (2007). Cerebrovascular disease and dementia. BJR 80 (special_issue_2), S121–7. 10.1259/bjr/75681080 18445742

[B158] Koch A. Ivers M. Gehrt A. Schnoor P. Rump A. Rieckert H. (2005). Cerebral autoregulation is temporarily disturbed in the early recovery phase after dynamic resistance exercise. Clin. Auton. Res. 15 (2), 83–91. 10.1007/s10286-005-0249-8 15834764

[B159] Komulainen P. Kivipelto M. Lakka T. A. Savonen K. Hassinen M. Kiviniemi V. (2010). Exercise, fitness and cognition – a randomised controlled trial in older individuals: the DR’s EXTRA study. Eur. Geriatr. Med. 1 (5), 266–72. 10.1016/j.eurger.2010.08.001

[B160] Korad S. Mündel T. Perry B. G. (2024). The effects of habitual resistance exercise training on cerebrovascular responses to lower body dynamic resistance exercise: a cross-sectional study. Exp. Physiol. 109, 1478–1491. 10.1113/EP091707 38888986 PMC11363110

[B161] Kovacevic A. Mavros Y. Heisz J. J. Fiatarone Singh M. A. (2018). The effect of resistance exercise on sleep: a systematic review of randomized controlled trials. Sleep. Med. Rev. 39, 52–68. 10.1016/j.smrv.2017.07.002 28919335

[B162] Kovacevic A. Fenesi B. Paolucci E. Heisz J. J. (2020). The effects of aerobic exercise intensity on memory in older adults. Appl. physiology, Nutr. metabolism 45 (6), 591–600. 10.1139/apnm-2019-0495 31665610

[B163] Kraemer W. J. Ratamess N. A. French D. N. (2002). Resistance training for health and performance. Curr. Sports Med. Rep. 1 (3), 165–71. 10.1249/00149619-200206000-00007 12831709

[B164] Kraemer W. J. Fragala M. S. Ratamess N. A. (2025). Evolution of resistance training in women: history and mechanisms for health and performance. Sports Med. Health Sci. 7, 351–365. 10.1016/j.smhs.2025.01.005 40936659 PMC12421175

[B165] Krizbai I. A. Bauer H. Bresgen N. Eckl P. M. Farkas A. Szatmári E. (2005). Effect of oxidative stress on the junctional proteins of cultured cerebral endothelial cells. Cell. Mol. Neurobiol. 25, 129–39. 10.1007/s10571-004-1378-7 15962510 PMC11529493

[B166] Kumar V. Kim S. H. Bishayee K. (2022). Dysfunctional glucose metabolism in alzheimer’s disease onset and potential pharmacological interventions. Int. J. Mol. Sci. 23 (17), 9540. 10.3390/ijms23179540 36076944 PMC9455726

[B167] Lachman M. E. Neupert S. D. Bertrand R. Jette A. M. (2006). The effects of strength training on memory in older adults. J. Aging Phys. Activity 14 (1), 59–73. 10.1123/japa.14.1.59 16648652

[B168] Landrigan J. F. Bell T. Crowe M. Clay O. J. Mirman D. (2020). Lifting cognition: a meta-analysis of effects of resistance exercise on cognition. Psychol. Res. 84 (5), 1167–83. 10.1007/s00426-019-01145-x 30627769

[B169] Laron Z. (2001). Insulin-like growth factor 1 (IGF-1): a growth hormone. Mol. Pathol. 54 (5), 311–316. 10.1136/mp.54.5.311 11577173 PMC1187088

[B170] Laufs U. Wassmann S. Czech T. Münzel T. Eisenhauer M. Böhm M. (2005). Physical inactivity increases oxidative stress, endothelial dysfunction, and atherosclerosis. Arteriosclerosis, thrombosis, Vasc. Biol. 25 (4), 809–14. 10.1161/01.ATV.0000158311.24443.af 15692095

[B171] Lee J. Kong S. Shin S. Lee G. Kim H. K. Shim Y. M. (2024). Wearable device–based intervention for promoting patient physical activity after lung cancer surgery: a nonrandomized clinical trial. JAMA Netw. Open 7 (9), e2434180. 10.1001/jamanetworkopen.2024.34180 39302678 PMC11415788

[B172] Lee J. West D. Pellegrini C. Wei J. Wilcox S. Neils-Strunjas J. (2025). Walking interventions and cognitive health in older adults: a systematic review of randomized controlled trials. Am. J. Health Promot, 08901171251328858. 10.1177/08901171251328858 40165427 PMC12284334

[B173] Lefferts W. K. Augustine J. A. Heffernan K. S. (2014). Effect of acute resistance exercise on carotid artery stiffness and cerebral blood flow pulsatility. Front. Physiol. 5, 101. 10.3389/fphys.2014.00101 24678301 PMC3958641

[B174] Lepley A. S. Hatzel B. M. (2010). Effects of weightlifting and breathing technique on blood pressure and heart rate. J. Strength and Cond. Res. 24 (8), 2179–2183. 10.1519/JSC.0b013e3181e2741d 20634749

[B175] Levinger I. Goodman C. Hare D. L. Jerums G. Selig S. (2007). The effect of resistance training on functional capacity and quality of life in individuals with high and low numbers of metabolic risk factors. Diabetes Care 30 (9), 2205–10. 10.2337/dc07-0841 17563342

[B176] Liao J. Wang J. Jia S. Cai Z. Liu H. (2024). Correlation of muscle strength, working memory, and activities of daily living in older adults. Front. Aging Neurosci. 16, 1453527. 10.3389/fnagi.2024.1453527 39372646 PMC11449751

[B177] Liguori G. American College of Sports Medicine (2020). ACSM’s guidelines for exercise testing and prescription. Lippincott williams and wilkins. 10.1249/JSR.0b013e31829a68cf 23851406

[B178] Lim J. Choi A. Kim B. (2024). The effects of resistance training on pain, strength, and function in osteoarthritis: systematic review and meta-analysis. J. Personalized Med. 14 (12), 1130. 10.3390/jpm14121130 39728043 PMC11676110

[B179] Liu Y. Chu J. M. T. Yan T. Zhang Y. Chen Y. Chang R. C. C. (2020). Short-term resistance exercise inhibits neuroinflammation and attenuates neuropathological changes in 3xTg Alzheimer’s disease mice. J. neuroinflammation 17, 4–16. 10.1186/s12974-019-1653-7 31900170 PMC6942350

[B180] Liu-Ambrose T. Nagamatsu L. S. Graf P. Beattie B. L. Ashe M. C. Handy T. C. (2010). Resistance training and executive functions: a 12-month randomized controlled trial. Archives Intern. Med. 170 (2), 170–8. 10.1001/archinternmed.2009.494 20101012 PMC3448565

[B181] Lommatzsch M. Zingler D. Schuhbaeck K. Schloetcke K. Zingler C. Schuff-Werner P. (2005). The impact of age, weight and gender on BDNF levels in human platelets and plasma. Neurobiol. aging 26 (1), 115–23. 10.1016/j.neurobiolaging.2004.03.002 15585351

[B182] Lopes K. G. Farinatti P. Bottino D. A. de Souza M. das G. C. Maranhão P. Bouskela E. (2022). Exercise with blood flow restriction improves muscle strength and mass while preserving the vascular and microvascular function and structure of older adults. Clin. Hemorheol. Microcirc. 82 (1), 13–26. 10.3233/CH-221395 35599474

[B183] Loprinzi P. D. (2016). Lower extremity muscular strength, sedentary behavior, and mortality. Age 38, 32–5. 10.1007/s11357-016-9899-9 26931822 PMC5005899

[B184] Loprinzi P. D. Frith E. Edwards M. K. (2018). Resistance exercise and episodic memory function: a systematic review. Clin. Physiology Funct. Imaging 38 (6), 923–9. 10.1111/cpf.12507 29368393

[B185] Lourenço C. F. Ledo A. Barbosa R. M. Laranjinha J. (2017). Neurovascular uncoupling in the triple transgenic model of Alzheimer’s disease: impaired cerebral blood flow response to neuronal-derived nitric oxide signaling. Exp. Neurol. 291, 36–43. 10.1016/j.expneurol.2017.01.013 28161255

[B186] Lu B. Nagappan G. Lu Y. (2014). “BDNF and synaptic plasticity, cognitive function, and dysfunction,” in Neurotrophic factors. Editors Lewin G. R. Carter B. D. (Berlin, Heidelberg: Springer Berlin Heidelberg), 223–50. 10.1007/978-3-642-45106-5_9 24668475

[B187] Lu A. Than S. Beare R. La Hood A. Collyer T. A. Srikanth V. (2024). Interactions between muscle volume and body mass index on brain structure in the UK biobank. Front. Dementia 3, 1456716. 10.3389/frdem.2024.1456716 39376216 PMC11456486

[B188] Lyle A. N. Raaz U. (2017). Killing me unsoftly: causes and mechanisms of arterial stiffness. Arteriosclerosis, Thrombosis, Vasc. Biol. 37 (2), e1–11. 10.1161/ATVBAHA.116.308563 28122777 PMC5308873

[B189] Macaulay T. R. Hegarty A. Yan L. Duncan D. Pa J. Kutch J. J. (2022). Effects of a 12-Week periodized Resistance training program on resting brain activity and cerebrovascular function: a nonrandomized pilot trial. Neurosci. insights 17 (101760670), 26331055221119441. 10.1177/26331055221119441 35983377 PMC9379950

[B190] MacDougall J. D. McKelvie R. S. Moroz D. E. Sale D. G. McCartney N. Buick F. (1992). Factors affecting blood pressure during heavy weight lifting and static contractions. J. Appl. Physiology 73 (4), 1590–7. 10.1152/jappl.1992.73.4.1590 1447109

[B191] Maldonado E. Morales-Pison S. Urbina F. Solari A. (2023). Aging hallmarks and the role of oxidative stress. Antioxidants 12 (3), 651. 10.3390/antiox12030651 36978899 PMC10044767

[B192] Malpas S. C. (2010). Sympathetic nervous system overactivity and its role in the development of cardiovascular disease. Physiol. Rev. 90 (2), 513–57. 10.1152/physrev.00007.2009 20393193

[B193] Marko D. M. Finch M. S. Mohammad A. MacNeil A. J. Klentrou P. MacPherson R. E. (2022). Postexercise serum from humans influences the biological tug of war of APP processing in human neuronal cells. Am. J. Physiology-Cell Physiology 322 (4), C614–23. 10.1152/ajpcell.00418.2021 35196169

[B194] Marston K. J. Newton M. J. Brown B. M. Rainey-Smith S. R. Bird S. Martins R. N. (2017). Intense resistance exercise increases peripheral brain-derived neurotrophic factor. J. Sci. Med. Sport 20 (10), 899–903. 10.1016/j.jsams.2017.03.015 28511848

[B195] Marston K. J. Peiffer J. J. Rainey-Smith S. R. Gordon N. Teo S. Y. Laws S. M. (2019). Resistance training enhances delayed memory in healthy middle-aged and older adults: a randomised controlled trial. J. Sci. Med. Sport 22 (11), 1226–31. 10.1016/j.jsams.2019.06.013 31281076

[B196] Mattsson N. Tosun D. Insel P. S. Simonson A. Jack C. R. Jr Beckett L. A. (2014). Association of brain amyloid-β with cerebral perfusion and structure in Alzheimer’s disease and mild cognitive impairment. Brain 137 (5), 1550–61. 10.1093/brain/awu043 24625697 PMC3999717

[B197] Mavros Y. Gates N. Wilson G. C. Jain N. Meiklejohn J. Brodaty H. (2017). Mediation of cognitive function improvements by strength gains after resistance training in older adults with mild cognitive impairment: Outcomes of the study of mental and resistance training. J. Am. Geriatrics Soc. 65 (3), 550–9. 10.1111/jgs.14542 28304092

[B198] Mayhan W. G. Arrick D. M. Sharpe G. M. Sun H. (2008). Age-related alterations in reactivity of cerebral arterioles: role of oxidative stress. Microcirculation 15 (3), 225–36. 10.1080/10739680701641421 18386218

[B199] Mcleod J. C. Currier B. S. Lowisz C. V. Phillips S. M. (2024). The influence of resistance exercise training prescription variables on skeletal muscle mass, strength, and physical function in healthy adults: an umbrella review. J. Sport Health Sci. 13 (1), 47–60. 10.1016/j.jshs.2023.06.005 37385345 PMC10818109

[B200] Meng S. J. Yu L. J. (2010). Oxidative stress, molecular inflammation and sarcopenia. Int. J. Mol. Sci. 11 (4), 1509–26. 10.3390/ijms11041509 20480032 PMC2871128

[B201] Meysami S. Raji C. A. Glatt R. M. Popa E. S. Ganapathi A. S. Bookheimer T. (2023). Handgrip strength is related to hippocampal and lobar brain volumes in a cohort of cognitively impaired older adults with confirmed amyloid burden. J. Alzheimer’s Dis. 91 (3), 999–1006. 10.3233/JAD-220886 36530088 PMC9912728

[B202] Mielke M. M. (2018). Sex and gender differences in Alzheimer’s disease dementia. Psychiatric times 35 (11), 14–17. 30820070 PMC6390276

[B203] Mitchell G. F. (1985)2008). Effects of central arterial aging on the structure and function of the peripheral vasculature: implications for end-organ damage. J. Appl. Physiol. 105 (5), 1652–60. 10.1152/japplphysiol.90549.2008 18772322 PMC2584844

[B204] Mitchell G. F. (2011). Aortic stiffness and cerebral blood flow. Am. J. Hypertens. 24, 1056. 10.1038/ajh.2011.112 21927008

[B205] Mitchell G. F. Hwang S. J. Vasan R. S. Larson M. G. Pencina M. J. Hamburg N. M. (2010). Arterial stiffness and cardiovascular events: the framingham heart study. Circulation 121 (4), 505–11. 10.1161/CIRCULATIONAHA.109.886655 20083680 PMC2836717

[B206] Miyachi M. (2013). Effects of resistance training on arterial stiffness: a meta-analysis. Br. J. Sports Med. 47 (6), 393–6. 10.1136/bjsports-2012-090488 22267567

[B207] Miyachi M. Kawano H. Sugawara J. Takahashi K. Hayashi K. Yamazaki K. (2004). Unfavorable effects of resistance training on central arterial compliance: a randomized intervention study. Circulation 110 (18), 2858–63. 10.1161/01.CIR.0000146380.08401.99 15492301

[B208] Mogensen F. L. Delle C. Nedergaard M. (2021). The glymphatic system (en)during inflammation. Int. J. Mol. Sci. 22 (14), 7491. 10.3390/ijms22147491 34299111 PMC8305763

[B209] Molina-Sotomayor E. Castillo-Quezada H. Martínez-Salazar C. González-Orb M. Espinoza-Salinas A. Gonzalez-Jurado J. A. (2020). Effects of progressive resistance training on cognition and igf-1 levels in elder women who live in areas with high air pollution. Int. J. Environ. Res. Public Health 17 (17), 1–17. 10.3390/ijerph17176203 32859109 PMC7503506

[B210] Moon Y. Moon W. J. Kim J. O. Kwon K. J. Han S. H. (2019). Muscle strength is independently related to brain atrophy in patients with alzheimer’s disease. Dementia Geriatric Cognitive Disord. 47 (4–6), 306–14. 10.1159/000500718 31311027

[B211] Morgan B. Mirza A. M. Gimblet C. J. Ortlip A. T. Ancalmo J. Kalita D. (2023). Effect of an 11-Week Resistance training program on arterial stiffness in young women. J. Strength Cond. Res. 37 (2), 315–21. 10.1519/JSC.0000000000004280 35916876

[B212] Moroni F. Ammirati E. Rocca M. A. Filippi M. Magnoni M. Camici P. G. (2018). Cardiovascular disease and brain health: focus on white matter hyperintensities. IJC Heart and Vasc. 19, 63–9. 10.1016/j.ijcha.2018.04.006 29946567 PMC6016077

[B213] Moyaert P. Padrela B. E. Morgan C. A. Petr J. Versijpt J. Barkhof F. (2023). Imaging blood-brain barrier dysfunction: a state-of-the-art review from a clinical perspective. Front. aging Neurosci. 15, 1132077. 10.3389/fnagi.2023.1132077 37139088 PMC10150073

[B214] Muhire G. Iulita M. F. Vallerand D. Youwakim J. Gratuze M. Petry F. R. (2019). Arterial stiffness due to carotid calcification disrupts cerebral blood flow regulation and leads to cognitive deficits. J. Am. Heart Assoc. 8 (9), e011630. 10.1161/JAHA.118.011630 31057061 PMC6512142

[B215] Murawska-Ciałowicz E. de Assis G. G. Clemente F. M. Feito Y. Stastny P. Zuwała-Jagiełło J. (2021). Effect of four different forms of high intensity training on BDNF response to wingate and graded exercise test. Sci. Rep. 11 (1), 8599. 10.1038/s41598-021-88069-y 33883635 PMC8060323

[B216] Nagamatsu L. S. Chan A. Davis J. C. Beattie B. L. Graf P. Voss M. W. (2013). Physical activity improves verbal and spatial memory in older adults with probable mild cognitive impairment: a 6‐month randomized controlled trial. J. aging Res. 2013 (1), 861893. 10.1155/2013/861893 23509628 PMC3595715

[B217] Nakamura N. Kubo T. Muraoka I. (2021). Effects of changes in large arterial compliance and small arterial buffer function with resistance training on cerebral blood flow pulsatility. Gazzetta Medica Ital. Arch. Sci. Mediche 180 (12), 805–14. 10.23736/s0393-3660.19.04300-6

[B218] Nelson M. E. Rejeski W. J. Blair S. N. Duncan P. W. Judge J. O. King A. C. (2007). Physical activity and public health in older adults: recommendation from the American college of sports medicine and the American heart association. Med. and Sci. Sports and Exerc. 39 (8), 1435–1445. 10.1249/mss.0b013e3180616aa2 17762378

[B219] Nelson A. R. Sweeney M. D. Sagare A. P. Zlokovic B. V. (2016). Neurovascular dysfunction and neurodegeneration in dementia and Alzheimer’s disease. Biochimica Biophysica Acta (BBA)-Molecular Basis Dis. 1862 (5), 887–900. 10.1016/j.bbadis.2015.12.016 26705676 PMC4821735

[B220] Newman A. B. Fitzpatrick A. L. Lopez O. Jackson S. Lyketsos C. Jagust W. (2005). Dementia and Alzheimer’s disease incidence in relationship to cardiovascular disease in the cardiovascular health study cohort. J. Am. Geriatrics Soc. 53 (7), 1101–7. 10.1111/j.1532-5415.2005.53360.x 16108925

[B221] Nicolakakis N. Hamel E. (2011). Neurovascular function in Alzheimer’s disease patients and experimental models. J. Cereb. Blood Flow and Metabolism 31 (6), 1354–70. 10.1038/jcbfm.2011.43 21468088 PMC3130325

[B222] Nieto-Bona M. P. García-Segura L. M. Torres-alemán I. (1997). Transynaptic modulation by insulin-like growth factor I of dendritic spines in purkinje cells. Int. J. Dev. Neurosci. 15 (6), 749–54. 10.1016/s0736-5748(97)00021-x 9402225

[B223] Nose D. Inoue H. Imaki K. Saku K. Miura S. ichiro (2023). Effects of a 14-week community health program of exercise and learning/education in older adults: a single-arm pre-post comparison study. Geriatr. Nurs. 51, 1–8. 10.1016/j.gerinurse.2023.02.012 36871326

[B224] Nouchi R. Taki Y. Takeuchi H. Sekiguchi A. Hashizume H. Nozawa T. (2014). Four weeks of combination exercise training improved executive functions, episodic memory, and processing speed in healthy elderly people: evidence from a randomized controlled trial. AGE 36 (2), 787–99. 10.1007/s11357-013-9588-x 24065294 PMC4039261

[B225] Okamoto T. Masuhara M. Ikuta K. (2006). Effects of eccentric and concentric resistance training on arterial stiffness. J. Hum. Hypertens. 20 (5), 348–54. 10.1038/sj.jhh.1001979 16496019

[B226] Okamoto T. Masuhara M. Ikuta K. (2009). Upper but not lower limb resistance training increases arterial stiffness in humans. Eur. J. Appl. Physiol. 107 (2), 127–34. 10.1007/s00421-009-1110-x 19533164

[B227] Ostrowski K. Rohde T. Asp S. Schjerling P. Pedersen B. K. (1999). Pro‐and anti‐inflammatory cytokine balance in strenuous exercise in humans. J. physiology 515 (1), 287–91. 10.1111/j.1469-7793.1999.287ad.x 9925898 PMC2269132

[B228] Özbeyli D. Sarı G. Özkan N. Karademir B. Yüksel M. Kaya Ö. T. Ç. (2017). Protective effects of different exercise modalities in an Alzheimer’s disease-like model. Behav. brain Res. 328, 159–77. 10.1016/j.bbr.2017.03.044 28390878

[B229] Ozturk E. D. Tan C. O. (2018). Human cerebrovascular function in health and disease: insights from integrative approaches. J. Physiological Anthropol. 37 (1), 4. 10.1186/s40101-018-0164-z 29454381 PMC5816507

[B230] O’Brien J T. Firbank M. J. Ritchie K. Wells K. Williams G. B. Ritchie C. W. (2020). Association between midlife dementia risk factors and longitudinal brain atrophy: the PREVENT-dementia study. J. Neurology, Neurosurg. and Psychiatry 91 (2), 158–61. 10.1136/jnnp-2019-321652 31806724

[B231] Paluch A. E. Boyer W. R. Franklin B. A. Laddu D. Lobelo F. Lee D. chul (2024). Resistance exercise training in individuals with and without cardiovascular disease: 2023 update: a scientific statement from the American heart association. Circulation 149 (3), e217–31. 10.1161/CIR.0000000000001189 38059362 PMC11209834

[B232] Pan W. Banks W. A. Fasold M. B. Bluth J. Kastin A. J. (1998). Transport of brain-derived neurotrophic factor across the blood–brain barrier. Neuropharmacology 37 (12), 1553–61. 10.1016/s0028-3908(98)00141-5 9886678

[B233] Pan L. Xie W. Fu X. Lu W. Jin H. Lai J. (2021). Inflammation and sarcopenia: a focus on circulating inflammatory cytokines. Exp. Gerontol. 154, 111544. 10.1016/j.exger.2021.111544 34478826

[B234] Park H. Poo M. ming (2013). Neurotrophin regulation of neural circuit development and function. Nat. Rev. Neurosci. 14 (1), 7–23. 10.1038/nrn3379 23254191

[B235] Patro S. Ratna S. Yamamoto H. A. Ebenezer A. T. Ferguson D. S. Kaur A. (2021). ATP synthase and mitochondrial bioenergetics dysfunction in Alzheimer’s disease. Int. J. Mol. Sci. 22 (20), 11185. 10.3390/ijms222011185 34681851 PMC8539681

[B236] Peig-Chiello P. Perrig W. J. Ehrsam R. Staehelin H. B. Krings F. (1998). The effects of resistance training on well-being and memory in elderly volunteers. Age Ageing 27 (4), 469–75. 10.1093/ageing/27.4.469 9884004

[B237] Peng S. L. Chen X. Li Y. Rodrigue K. M. Park D. C. Lu H. (2018). Age-related changes in cerebrovascular reactivity and their relationship to cognition: a four-year longitudinal study. NeuroImage 174, 257–62. 10.1016/j.neuroimage.2018.03.033 29567504 PMC5949266

[B238] Pereira D. S. De Queiroz B. Z. Miranda A. S. Rocha N. P. Felício D. C. Mateo E. C. (2013). Effects of physical exercise on plasma levels of brain-derived neurotrophic factor and depressive symptoms in elderly Women—A randomized clinical trial. Archives Phys. Med. rehabilitation 94 (8), 1443–50. 10.1016/j.apmr.2013.03.029 23602881

[B239] Perrone C. E. Fenwick-Smith D. Vandenburgh H. H. (1995). Collagen and stretch modulate autocrine secretion of insulin-like growth Factor-1 and insulin-like growth factor binding proteins from differentiated skeletal muscle cells (∗). J. Biol. Chem. 270 (5), 2099–106. 10.1074/jbc.270.5.2099 7530717

[B240] Perry B. G. Lucas S. J. E. (2021). The acute cardiorespiratory and cerebrovascular response to resistance exercise. Sports Med. - Open 7 (1), 36. 10.1186/s40798-021-00314-w 34046740 PMC8160070

[B241] Phillips A. A. Chan F. H. Zheng M. M. Z. Krassioukov A. V. Ainslie P. N. (2016). Neurovascular coupling in humans: physiology, methodological advances and clinical implications. J. Cereb. Blood Flow. Metab. 36 (4), 647–64. 10.1177/0271678X15617954 26661243 PMC4821024

[B242] Planche V. Coupé P. Helmer C. Le Goff M. Amieva H. Tison F. (2019). Evolution of brain atrophy subtypes during aging predicts long-term cognitive decline and future Alzheimer’s clinical syndrome. Neurobiol. aging 79, 22–9. 10.1016/j.neurobiolaging.2019.03.006 31026619

[B243] Protas H. D. Chen K. Langbaum J. B. Fleisher A. S. Alexander G. E. Lee W. (2013). Posterior cingulate glucose metabolism, hippocampal glucose metabolism, and hippocampal volume in cognitively normal, late-middle-aged persons at 3 levels of genetic risk for alzheimer disease. JAMA neurol. 70 (3), 320–5. 10.1001/2013.jamaneurol.286 23599929 PMC3745014

[B244] Pulford B. E. Ishii D. N. (2001). Uptake of circulating insulin-like growth factors (IGFs) into cerebrospinal fluid appears to be independent of the IGF receptors as well as IGF-Binding proteins. Endocrinology 142 (1), 213–20. 10.1210/endo.142.1.7894 11145584

[B245] Qian F. Huo D. (2020). Circulating insulin-like growth Factor-1 and risk of total and 19 site-specific cancers: Cohort study analyses from the UK biobank. Cancer Epidemiol. Biomarkers and Prev. 29 (11), 2332–42. 10.1158/1055-9965.EPI-20-0743 32856611 PMC7642199

[B246] Rajan K. B. Weuve J. Barnes L. L. McAninch E. A. Wilson R. S. Evans D. A. (2021). Population estimate of people with clinical Alzheimer’s disease and mild cognitive impairment in the United States (2020–2060). Alzheimer’s and Dementia 17 (12), 1966–75. 10.1002/alz.12362 34043283 PMC9013315

[B247] Ramírez-Vélez R. Castro-Astudillo K. Correa-Bautista J. E. González-Ruíz K. Izquierdo M. García-Hermoso A. (2020). The effect of 12 weeks of different exercise training modalities or nutritional guidance on cardiometabolic risk factors, vascular parameters, and physical fitness in overweight adults: cardiometabolic high-intensity interval training-resistance training randomized controlled study. J. Strength and Cond. Res. 34 (8), 2178–2188. 10.1519/JSC.0000000000003533 32187150

[B248] Ramos-Campo D. J. Scott B. R. Alcaraz P. E. Rubio-Arias J. A. (2018). The efficacy of resistance training in hypoxia to enhance strength and muscle growth: a systematic review and meta-analysis. Eur. J. sport Sci. 18 (1), 92–103. 10.1080/17461391.2017.1388850 29045191

[B249] Rasmussen P. Brassard P. Adser H. Pedersen M. V. Leick L. Hart E. (2009). Evidence for a release of brain-derived neurotrophic factor from the brain during exercise. Exp. Physiol. 94 (10), 1062–9. 10.1113/expphysiol.2009.048512 19666694

[B250] Reddy O. C. van der Werf Y. D. (2020). The sleeping brain: harnessing the power of the glymphatic system through lifestyle choices. Brain Sci. 10 (11), 868. 10.3390/brainsci10110868 33212927 PMC7698404

[B251] Roher A. E. Garami Z. Tyas S. L. Maarouf C. L. Kokjohn T. A. Belohlavek M. (2011). Transcranial doppler ultrasound blood flow velocity and pulsatility index as systemic indicators for Alzheimer’s disease. Alzheimer’s Dement. 7 (4), 445–55. 10.1016/j.jalz.2010.09.002 21388892 PMC3117072

[B252] Rojas‐Gutierrez E. Muñoz‐Arenas G. Treviño S. Espinosa B. Chavez R. Rojas K. (2017). Alzheimer’s disease and metabolic syndrome: a link from oxidative stress and inflammation to neurodegeneration. Synapse 71 (10), e21990. 10.1002/syn.21990 28650104

[B253] Rondão C. A. de M. Mota M. P. Oliveira M. M. Peixoto F. Esteves D. (2022). Multicomponent exercise program effects on fitness and cognitive function of elderlies with mild cognitive impairment: involvement of oxidative stress and BDNF. Front. Aging Neurosci. 14, 950937. 10.3389/fnagi.2022.950937 36092805 PMC9453672

[B254] Rossow L. M. Fahs C. A. Thiebaud R. S. Loenneke J. P. Kim D. Mouser J. G. (2014). Arterial stiffness and blood flow adaptations following eight weeks of resistance exercise training in young and older women. Exp. Gerontol. 53, 48–56. 10.1016/j.exger.2014.02.010 24566193

[B255] Roy M. A. Labrecque L. Perry B. G. Korad S. Smirl J. D. Brassard P. (2022). Directional sensitivity of the cerebral pressure-flow relationship in young healthy individuals trained in endurance and resistance exercise. Exp. Physiol. 107 (4), 299–311. 10.1113/EP090159 35213765

[B256] Ruiz J. R. Gil-Bea F. Bustamante-Ara N. Rodríguez-Romo G. Fiuza-Luces C. Serra-Rexach J. A. (2015). Resistance training does not have an effect on cognition or related serum biomarkers in nonagenarians: a randomized controlled trial. Int. J. Sports Med. 36 (1), 54–60. 10.1055/s-0034-1375693 25329433

[B257] Sabayan B. Jansen S. Oleksik A. M. van Osch M. J. P. van Buchem M. A. van Vliet P. (2012). Cerebrovascular hemodynamics in Alzheimer’s disease and vascular dementia: a meta-analysis of transcranial doppler studies. Ageing Res. Rev. 11 (2), 271–7. 10.1016/j.arr.2011.12.009 22226802

[B258] Safar M. E. (2018). Arterial stiffness as a risk factor for clinical hypertension. Nat. Rev. Cardiol. 15 (2), 97–105. 10.1038/nrcardio.2017.155 29022570

[B259] Sagiv M. (2009). Safety of resistance training in the elderly. Eur. Rev. Aging Phys. Activity 6 (1), 1–2. 10.1007/s11556-009-0047-8

[B260] Sartorius A. Hellweg R. Litzke J. Vogt M. Dormann C. Vollmayr B. (2009). Correlations and discrepancies between serum and brain tissue levels of neurotrophins after electroconvulsive treatment in rats. Pharmacopsychiatry 42 (06), 270–6. 10.1055/s-0029-1224162 19924587

[B261] Schimidt H. L. Garcia A. Izquierdo I. Mello-Carpes P. B. Carpes F. P. (2019). Strength training and running elicit different neuroprotective outcomes in a β-amyloid peptide-mediated Alzheimer’s disease model. Physiology Behav. 206, 206–12. 10.1016/j.physbeh.2019.04.012 30995451

[B262] Shah T. Verdile G. Sohrabi H. Campbell A. Putland E. Cheetham C. (2014). A combination of physical activity and computerized brain training improves verbal memory and increases cerebral glucose metabolism in the elderly. Transl. Psychiatry 4 (12), e487. 10.1038/tp.2014.122 25463973 PMC4270308

[B263] Shaito A. Aramouni K. Assaf R. Parenti A. Orekhov A. Yazbi A. E. (2022). Oxidative stress-induced endothelial dysfunction in cardiovascular diseases. FBL 27 (3), 105–null. 10.31083/j.fbl2703105 35345337

[B264] Shaughnessy K. A. Hackney K. J. Clark B. C. Kraemer W. J. Terbizan D. J. Bailey R. R. (2020). A narrative review of handgrip strength and cognitive functioning: bringing a new characteristic to muscle memory. J. Alzheimer’s Dis. 73 (4), 1265–78. 10.3233/JAD-190856 31929158 PMC7063546

[B265] Silva JKTNF Menêses A. L. Parmenter B. J. Ritti-Dias R. M. Farah B. Q. (2021). Effects of resistance training on endothelial function: a systematic review and meta-analysis. Atherosclerosis 333, 91–9. 10.1016/j.atherosclerosis.2021.07.009 34399984

[B266] Silveira-Rodrigues J. G. Nogueira N. G. de H. M. Faria L. O. Pereira D. S. Soares D. D. (2023). Combined training improves executive functions without changing brain-derived neurotrophic factor levels of middle-aged and older adults with type 2 diabetes. Exp. Clin. Endocrinol. and Diabetes 131 (06), 345–53. 10.1055/a-2069-4050 37019176

[B267] Silvestrini M. Pasqualetti P. Baruffaldi R. Bartolini M. Handouk Y. Matteis M. (2006). Cerebrovascular reactivity and cognitive decline in patients with alzheimer disease. Stroke 37 (4), 1010–5. 10.1161/01.STR.0000206439.62025.97 16497984

[B268] Sims-Robinson C. Kim B. Feldman E. L. (2015). “Diabetes and cognitive dysfunction,” in Neurobiology of brain disorders Elsevier, 189–201.

[B269] Singer J. Trollor J. N. Baune B. T. Sachdev P. S. Smith E. (2014). Arterial stiffness, the brain and cognition: a systematic review. Ageing Res. Rev. 15, 16–27. 10.1016/j.arr.2014.02.002 24548924

[B270] Steensberg A. Keller C. Starkie R. L. Osada T. Febbraio M. A. Pedersen B. K. (2002). IL-6 and TNF-α expression in, and release from, contracting human skeletal muscle. Am. J. Physiology-Endocrinology Metabolism 283 (6), E1272–8. 10.1152/ajpendo.00255.2002 12388119

[B271] Stefanidis K. B. Askew C. D. Greaves K. Summers M. J. (2018). The effect of non-stroke cardiovascular disease states on risk for cognitive decline and dementia: a systematic and meta-analytic review. Neuropsychol. Rev. 28, 1–15. 10.1007/s11065-017-9359-z 28856507

[B272] Stimson A. M. Anderson C. Holt A. M. Henderson A. J. (2024). Why don’t women engage in muscle strength exercise? An integrative review. Health Promot. J. Aust. 35 (4), 911–23. 10.1002/hpja.857 38566279

[B273] Suo C. Singh M. F. Gates N. Wen W. Sachdev P. Brodaty H. (2016). Therapeutically relevant structural and functional mechanisms triggered by physical and cognitive exercise. Mol. Psychiatry 21 (11), 1633–42. 10.1038/mp.2016.19 27001615 PMC5078857

[B274] Suri S. Chiesa S. T. Zsoldos E. Mackay C. E. Filippini N. Griffanti L. (2020). Accelerated aortic stiffness is associated with brain structure, perfusion and cognition in the whitehall II imaging Sub-study. Cardiovasc. Med. 10.1371/journal.pmed.1003467 33373359 PMC7771705

[B275] Sweeney M. D. Kisler K. Montagne A. Toga A. W. Zlokovic B. V. (2018a). The role of brain vasculature in neurodegenerative disorders. Nat. Neurosci. 21 (10), 1318–31. 10.1038/s41593-018-0234-x 30250261 PMC6198802

[B276] Sweeney M. D. Sagare A. P. Zlokovic B. V. (2018b). Blood–brain barrier breakdown in alzheimer disease and other neurodegenerative disorders. Nat. Rev. Neurol. 14 (3), 133–50. 10.1038/nrneurol.2017.188 29377008 PMC5829048

[B277] Swift D. L. Johannsen N. M. Myers V. H. Earnest C. P. Smits J. A. J. Blair S. N. (2012). The effect of exercise training modality on serum brain derived neurotrophic factor levels in individuals with type 2 diabetes. PLoS ONE 7 (8), e42785. 10.1371/journal.pone.0042785 22880108 PMC3412800

[B278] ten Brinke L. F. Bolandzadeh N. Nagamatsu L. S. Hsu C. L. Davis J. C. Miran-Khan K. (2015). Aerobic exercise increases hippocampal volume in older women with probable mild cognitive impairment: a 6-month randomised controlled trial. Br. J. Sports Med. 49 (4), 248–254. 10.1136/bjsports-2013-093184 24711660 PMC4508129

[B279] Ten Kate M. Dicks E. Visser P. J. van der Flier W. M. Teunissen C. E. Barkhof F. (2018). Atrophy subtypes in prodromal Alzheimer’s disease are associated with cognitive decline. Brain 141 (12), 3443–56. 10.1093/brain/awy264 30351346 PMC6669409

[B280] Thapa N. Yang J. G. Bae S. Kim G. M. Park H. J. Park H. (2023). Effect of electrical muscle stimulation and resistance exercise intervention on physical and brain function in middle-aged and older women. Int. J. Environ. Res. Public Health 20 (1), 101. 10.3390/ijerph20010101 36612423 PMC9819342

[B281] Thomas B. P. Tarumi T. Sheng M. Tseng B. Womack K. B. Cullum C. M. (2020). Brain perfusion change in patients with mild cognitive impairment after 12 months of aerobic exercise training. J. Alzheimer’s Dis. 75 (2), 617–31. 10.3233/JAD-190977 32310162 PMC8062932

[B282] Thomas H. J. Marsh C. E. Naylor L. H. Ainslie P. N. Smith K. J. Carter H. H. (2021). Resistance, but not endurance exercise training, induces changes in cerebrovascular function in healthy young subjects. Am. J. Physiol. Heart Circ. Physiol. 321 (5), H881–92. 10.1152/ajpheart.00230.2021 34559581

[B283] Tomoto T. Verma A. Kostroske K. Tarumi T. Patel N. R. Pasha E. P. (2022). One-year aerobic exercise increases cerebral blood flow in cognitively normal older adults. J. Cereb. Blood Flow. Metab. 43, 404–418. 10.1177/0271678X221133861 36250505 PMC9941859

[B284] Törpel A. Herold F. Hamacher D. Müller N. G. Schega L. (2018). Strengthening the brain—is resistance training with blood flow restriction an effective strategy for cognitive improvement? J. Clin. Med. 7 (10), 337. 10.3390/jcm7100337 30304785 PMC6210989

[B285] Tsai C. L. Wang C. H. Pan C. Y. Chen F. C. Huang T. H. Chou F. Y. (2014). Executive function and endocrinological responses to acute resistance exercise. Front. Behav. Neurosci. 8 (AUG), 262. 10.3389/fnbeh.2014.00262 25136300 PMC4117935

[B286] Tsai C. L. Ukropec J. Ukropcová B. Pai M. C. (2018). An acute bout of aerobic or strength exercise specifically modifies circulating exerkine levels and neurocognitive functions in elderly individuals with mild cognitive impairment. NeuroImage Clin. 17, 272–84. 10.1016/j.nicl.2017.10.028 29527475 PMC5842646

[B287] Tsao C. W. Seshadri S. Beiser A. S. Westwood A. J. Decarli C. Au R. (2013). Relations of arterial stiffness and endothelial function to brain aging in the community. Neurology 81 (11), 984–91. 10.1212/WNL.0b013e3182a43e1c 23935179 PMC3888200

[B288] Tsutsumi T. Don B. M. Zaichkowsky L. D. Delizonna L. L. (1997). Physical fitness and psychological benefits of strength training in community dwelling older adults. Appl. Hum. Sci. 16 (6), 257–66. 10.2114/jpa.16.257 9545677

[B289] Vaishya R. Misra A. Vaish A. Ursino N. D’Ambrosi R. (2024). Hand grip strength as a proposed new vital sign of health: a narrative review of evidences. J. Health, Popul. Nutr. 43 (1), 7. 10.1186/s41043-024-00500-y 38195493 PMC10777545

[B290] Van Dam R. Van Ancum J. M. Verlaan S. Scheerman K. Meskers C. G. Maier A. B. (2018). Lower cognitive function in older patients with lower muscle strength and muscle mass. Dementia geriatric cognitive Disord. 45 (3–4), 243–50. 10.1159/000486711 29913450 PMC6067649

[B291] van Dijk S. E. Drenth N. Hafkemeijer A. Labadie G. Witjes-Ané M. N. W. Blauw G. J. (2024). Neurovascular coupling in early stage dementia – a case-control study. J. Cereb. Blood Flow. Metab. 44 (6), 1013–23. 10.1177/0271678X231214102 37994030 PMC11318393

[B292] Vaughan S. wallis M. polit D. steele M. shum D. Morris N. (2014). The effects of multimodal exercise on cognitive and physical functioning and brain-derived neurotrophic factor in older women: a randomised controlled trial. Age Ageing 43 (5), 623–9. 10.1093/ageing/afu010 24554791

[B293] Veen J. Montiel-Rojas D. Nilsson A. Kadi F. (2021). Engagement in muscle-strengthening activities lowers sarcopenia risk in older adults already adhering to the aerobic physical activity guidelines. Int. J. Environ. Res. public health 18 (3), 989. 10.3390/ijerph18030989 33499423 PMC7908493

[B294] Vega S. R. Knicker A. Hollmann W. Bloch W. Strüder H. K. (2010). Effect of resistance exercise on serum levels of growth factors in humans. Hormone metabolic Res. 42 (13), 982–6. 10.1055/s-0030-1267950 21053157

[B295] Vermeer S. E. Rinkel G. J. Algra A. (1997). Circadian fluctuations in onset of subarachnoid hemorrhage: new data on aneurysmal and perimesencephalic hemorrhage and a systematic review. Stroke 28 (4), 805–8. 10.1161/01.str.28.4.805 9099200

[B296] Vicenzini E. Ricciardi M. C. Altieri M. Puccinelli F. Bonaffini N. Di P. V. (2007). Cerebrovascular reactivity in degenerative and vascular dementia: a transcranial doppler study. Eur. Neurol. 58 (2), 84–9. 10.1159/000103642 17565221

[B297] Vilela T. C. Muller A. P. Damiani A. P. Macan T. P. da Silva S. Canteiro P. B. (2017). Strength and aerobic exercises improve spatial memory in aging rats through stimulating distinct neuroplasticity mechanisms. Mol. Neurobiol. 54, 7928–37. 10.1007/s12035-016-0272-x 27878552

[B298] Vints W. A. J. Šeikinaitė J. Gökçe E. Kušleikienė S. Šarkinaite M. Valatkeviciene K. (2024). Resistance exercise effects on hippocampus subfield volumes and biomarkers of neuroplasticity and neuroinflammation in older adults with low and high risk of mild cognitive impairment: a randomized controlled trial. GeroScience 46 (4), 3971–91. 10.1007/s11357-024-01110-6 38478179 PMC11226571

[B299] Walsh J. J. Scribbans T. D. Bentley R. F. Mikhail Kellawan J. Gurd B. Tschakovsky M. E. (2015). Neurotrophic growth factor responses to lower body resistance training in older adults. Appl. Physiology, Nutr. Metabolism 41 (3), 315–23. 10.1139/apnm-2015-0410 26886517

[B300] Wang J. Gu B. J. Masters C. L. Wang Y. J. (2017). A systemic view of alzheimer disease — insights from amyloid-β metabolism beyond the brain. Nat. Rev. Neurol. 13 (10), 703–23. 10.1038/nrneurol.2017.147 29027541

[B301] Wang C. Reid G. Mackay C. E. Hayes G. Bulte D. P. Suri S. (2023). A systematic review of the association between dementia risk factors and cerebrovascular reactivity. Neurosci. and Biobehav. Rev. 148, 105140. 10.1016/j.neubiorev.2023.105140 36944391

[B302] Westcott W. L. (2012). Resistance training is medicine: effects of strength training on health. Curr. Sports Med. Rep. 11 (4), 209–216. 10.1249/JSR.0b013e31825dabb8 22777332

[B303] Westwood A. J. Beiser A. DeCarli C. Harris T. B. Chen T. C. mei He X. (2014). Insulin-like growth factor-1 and risk of alzheimer dementia and brain atrophy. Neurology 82 (18), 1613–9. 10.1212/WNL.0000000000000382 24706014 PMC4013812

[B304] Williams M. A. Haskell W. L. Ades P. A. Amsterdam E. A. Bittner V. Franklin B. A. (2007). Resistance exercise in individuals with and without cardiovascular disease: 2007 update: a scientific statement from the American heart association council on clinical cardiology and council on nutrition, physical activity, and metabolism. Circulation 116 (5), 572–84. 10.1161/CIRCULATIONAHA.107.185214 17638929

[B305] Willie C. K. Tzeng Y. C. Fisher J. A. Ainslie P. N. (2014). Integrative regulation of human brain blood flow. J. Physiol. (Lond). 592 (5), 841–59. 10.1113/jphysiol.2013.268953 24396059 PMC3948549

[B306] Wolters F. J. Zonneveld H. I. Hofman A. van der Lugt A. Koudstaal P. J. Vernooij M. W. (2017). Cerebral perfusion and the risk of dementia: a population-based study. Circulation 136 (8), 719–28. 10.1161/CIRCULATIONAHA.117.027448 28588075

[B307] Xie L. Kang H. Xu Q. Chen M. J. Liao Y. Thiyagarajan M. (2013). Sleep drives metabolite clearance from the adult brain. science. 342 (6156), 373–7. 10.1126/science.1241224 24136970 PMC3880190

[B308] Xu X. Jerskey B. A. Cote D. M. Walsh E. G. Hassenstab J. J. Ladino M. E. (2014). Cerebrovascular perfusion among older adults is moderated by strength training and gender. Neurosci. Lett. 560, 26–30. 10.1016/j.neulet.2013.12.011 24355360 PMC3920729

[B309] Yamaguchi S. Meguro K. Itoh M. Hayasaka C. Shimada M. Yamazaki H. (1997). Decreased cortical glucose metabolism correlates with hippocampal atrophy in Alzheimer’s disease as shown by MRI and PET. J. Neurology, Neurosurg. and Psychiatry 62 (6), 596–600. 10.1136/jnnp.62.6.596 9219745 PMC1074143

[B310] Yang L. Yan Y. Wang Y. Hu X. Lu J. Chan P. (2018). Gradual disturbances of the amplitude of low-frequency fluctuations (ALFF) and fractional ALFF in alzheimer spectrum. Front. Neurosci. 12, 975. 10.3389/fnins.2018.00975 30618593 PMC6306691

[B311] Yarrow J. F. White L. J. McCoy S. C. Borst S. E. (2010). Training augments resistance exercise induced elevation of circulating brain derived neurotrophic factor (BDNF). Neurosci. Lett. 479 (2), 161–5. 10.1016/j.neulet.2010.05.058 20553806

[B312] Yew B. Nation D. A. for the Alzheimer’s Disease Neuroimaging Initiative (2017). Cerebrovascular resistance: effects on cognitive decline, cortical atrophy, and progression to dementia. Brain 140 (7), 1987–2001. 10.1093/brain/awx112 28575149 PMC6059092

[B313] Yin F. Sancheti H. Patil I. Cadenas E. (2016). Energy metabolism and inflammation in brain aging and Alzheimer’s disease. Free Radic. Biol. Med. 100, 108–22. 10.1016/j.freeradbiomed.2016.04.200 27154981 PMC5094909

[B314] Yoon D. H. Kang D. Kim H. jae Kim J. S. Song H. S. Song W. (2017). Effect of elastic band-based high-speed power training on cognitive function, physical performance and muscle strength in older women with mild cognitive impairment. Geriatrics and Gerontology Int. 17 (5), 765–72. 10.1111/ggi.12784 27396580

[B315] Zagrebelsky M. Korte M. (2014). Form follows function: BDNF and its involvement in sculpting the function and structure of synapses. Neuropharmacology 76, 628–38. 10.1016/j.neuropharm.2013.05.029 23752094

[B316] Zhang B. Lin L. Wu S. (2021a). A review of brain atrophy subtypes definition and analysis for Alzheimer’s disease heterogeneity studies. J. Alzheimer’s Dis. 80 (4), 1339–52. 10.3233/JAD-201274 33682711

[B317] Zhang H. Wang Y. Lyu D. Li Y. Li W. Wang Q. (2021b). Cerebral blood flow in mild cognitive impairment and Alzheimer’s disease: a systematic review and meta-analysis. Ageing Res. Rev. 71, 101450. 10.1016/j.arr.2021.101450 34419673

[B318] Zhang Y. Zhang Y. J. Zhang H. W. Ye W. B. Korivi M. (2021c). Low-to-moderate-intensity resistance exercise is more effective than high-intensity at improving endothelial function in adults: a systematic review and meta-analysis. Int. J. Environ. Res. Public Health 18 (13), 6723. 10.3390/ijerph18136723 34206463 PMC8297299

[B319] Zhang T. Tian G. Wang X. (2022). Effects of low-load blood flow restriction training on hemodynamic responses and vascular function in older adults: a meta-analysis. Int. J. Environ. Res. Public Health 19 (11), 6750. 10.3390/ijerph19116750 35682336 PMC9180641

[B320] Zhao H. Cheng R. Song G. Teng J. Shen S. Fu X. (2022). The effect of resistance training on the rehabilitation of elderly patients with sarcopenia: a meta-analysis. Int. J. Environ. Res. Public Health 19 (23), 15491. 10.3390/ijerph192315491 36497565 PMC9739568

[B321] Zhou Y. Wu W. Zou Y. Huang W. Lin S. Ye J. (2022). Benefits of different combinations of aerobic and resistance exercise for improving plasma glucose and lipid metabolism and sleep quality among elderly patients with metabolic syndrome: a randomized controlled trial. Endocr. J. 69 (7), 819–30. 10.1507/endocrj.EJ21-0589 35197411

[B322] Ziegenhorn A. A. Schulte-Herbrüggen O. Danker-Hopfe H. Malbranc M. Hartung H. D. Anders D. (2007). Serum Neurotrophins—A study on the time course and influencing factors in a large old age sample. Neurobiol. aging 28 (9), 1436–45. 10.1016/j.neurobiolaging.2006.06.011 16879899

[B323] Zlokovic B. V. (2004). Clearing amyloid through the blood–brain barrier. J. Neurochem. 89 (4), 807–11. 10.1111/j.1471-4159.2004.02385.x 15140180

[B324] Zlokovic B. V. (2005). Neurovascular mechanisms of Alzheimer’s neurodegeneration. Trends Neurosci. 28 (4), 202–8. 10.1016/j.tins.2005.02.001 15808355

[B325] Zoeller R. F. Angelopoulos T. J. Thompson B. C. Wenta M. R. Price T. B. Thompson P. D. (2009). Vascular remodeling in response to 12 wk of upper arm unilateral resistance training. Med. Sci. sports Exerc. 41 (11), 2003–2008. 10.1249/MSS.0b013e3181a70707 19812518 PMC4107658

